# A New Parametric Accelerated Failure Time Model for Semi‐Competing Risks Data

**DOI:** 10.1002/sim.70405

**Published:** 2026-04-06

**Authors:** Antoniya Dineva, Oliver Kuss, Annika Hoyer

**Affiliations:** ^1^ Biostatistics and Medical Biometry, Medical School OWL Bielefeld University Bielefeld Germany; ^2^ German Diabetes Center, Institute for Biometrics and Epidemiology Leibniz Center for Diabetes Research at Heinrich Heine University Düsseldorf Düsseldorf Germany

**Keywords:** cohort studies, competing risks, time‐to‐event model, truncated data

## Abstract

In cohort studies, the occurrence of a specific disease is often of main interest. For proper modeling, death as a competing risk should be accounted for. Especially, the “semi‐competing” character of the data has to be acknowledged: Disease occurrence (the non‐terminal event) can be observed before death (the terminal event), but not vice versa. Hence, the terminal event might censor the non‐terminal event but remains observable if the non‐terminal event occurs first. The underlying setting can be described by an illness‐death model incorporating transitions between the three states “healthy,” “diseased,” and “dead.” This article aims to introduce a new method employing accelerated failure time (AFT) models for each of the three transitions between the states of the illness‐death model. The major advantage of this approach is its intuitive and straightforward interpretation based on the survival instead of the hazard function, facilitating communication of results. We propose a parametric model by assuming Weibull distributions for the ages in the different states, modeling (1) the age at disease onset, (2) the age at death for patients without diagnosis, and (3) the age at death after diagnosis. We add random effects to adjust for intra‐individual correlations, yielding a trivariate model. Model parameters are estimated by the maximum likelihood principle. The likelihood function incorporates left truncation for both terminal and non‐terminal events, reflecting the delayed entry into the study cohort. As in cohort studies the diagnosis is usually made at intermittent follow‐up visits and the exact age at disease onset is only known to lie in the interval between the last two visits, we finally consider interval censoring for the disease occurrence. The model is illustrated using data from the PAQUID study, focusing on dementia as a non‐terminal event. Our approach leads to plausible results, confirming the findings by other existing methods. The overall performance is assessed by a simulation study, yielding promising results concerning the accuracy and numerical robustness of our model.

## 1 Introduction

Bivariate time‐to‐event outcomes are frequently observed in cohort studies where participants are followed up for the occurrence of a particular disease (the non‐terminal event). However, death (the terminal event) might occur first and censor the disease onset, but not vice versa. This specific setting is often referred to as *semi‐competing risk* data [1]. Unlike the classical competing risks in which individuals are susceptible to several types of events but can experience only one, in the semi‐competing risks framework both events might be observable for the same subject. To accurately model the joint distribution of the two outcomes and their underlying dependencies, the application of methods for semi‐competing risks is essential. In general, these methods can be divided into at least two main groups including copula‐based models and the illness‐death model (IDM) (Table 1). The common approach of copula‐based methods is to define a two‐variate copula function to model the joint distribution of the age at the terminal and non‐terminal events, considering the dependency between them. In a recent publication, Wei et al. [11] developed a conditional copula model incorporating four different copula functions and allowing both, the association parameter between the time‐to‐event endpoints and the hazard ratios, to depend on binary covariates. Parametric assumptions were made for the marginal distributions of the event times. In another recently published article, Sun et al. [13] proposed a semi‐parametric copula model, incorporating interval censoring for the non‐terminal event and left truncation due to the delayed entry into the study cohort. However, the application of a copula model does not allow any statements on people who die with a diagnosed disease as only the general distribution of the age at death is estimated.

**TABLE 1:** Overview of existing methods for semi‐competing risks data.

1. Copula‐based models	2. IDM methods	
	2.1. PH models	2.2. AFT models
Peng and Fine 2007 [2]	Xu et al. 2010 [3]	Lee et al. 2017 [4]
Lakhal et al. 2008 [5]	Lee et al. 2015 [6]	Kats and Gorfine 2022 [7]
Hsieh et al. 2008 [8]	Gorfine et al. 2021 [9]	Zamsheva et al. 2024 [10]
Wei et al. 2023 [11]	Lee et al. 2021 [12]	
Sun et al. 2023 [13]		

**FIGURE 1:**
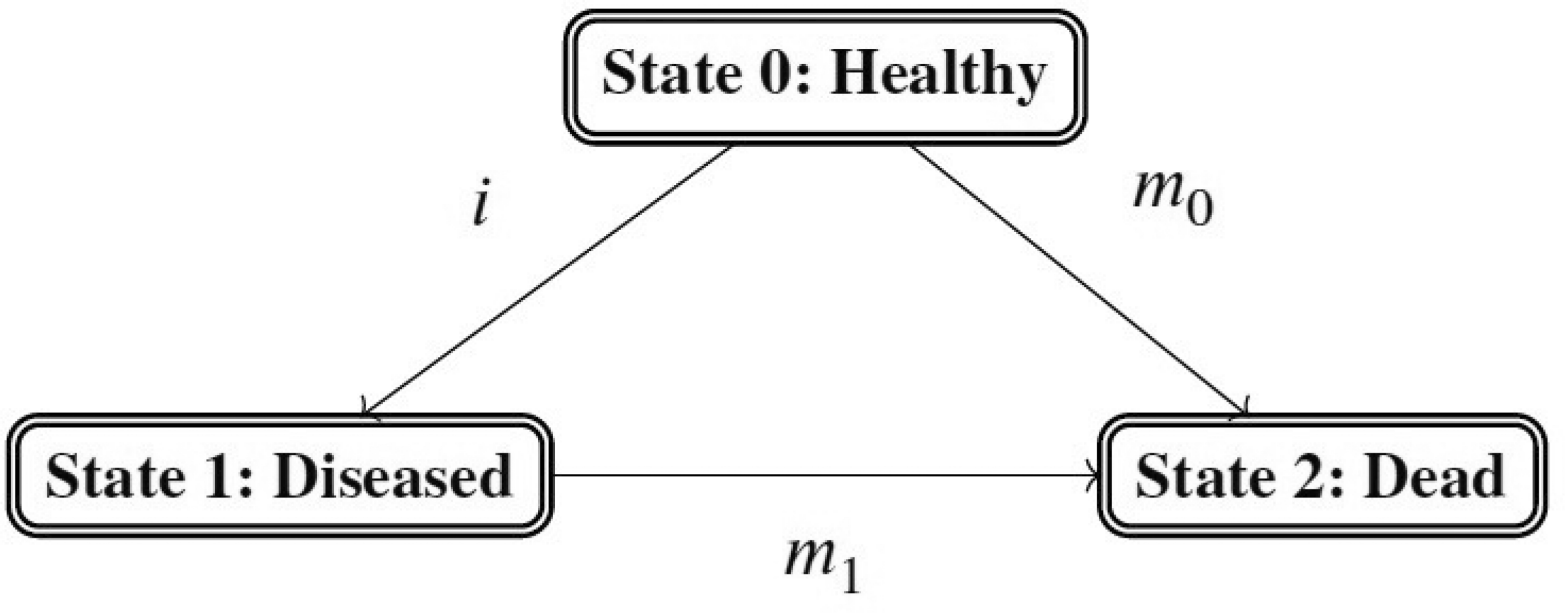
Illness‐death model with three states: Healthy, diseased, and dead and three transitions: Incidence i, mortality without disease $m_{0}$, and mortality with disease $m_{1}$.

The second group includes methods based on the IDM that incorporate the transitions between the three states “healthy” (with respect to the disease of interest), “diseased,” and “dead” (Figure 1). In the IDM, each individual starts in the “healthy” state and then moves either to the “diseased” state (described by the incidence i) in case of diagnosis, or dies and moves to the state “dead” (described by the mortality $m_{0}$). It is furthermore important to consider the transition between the states “diseased” and “dead” (described by the mortality $m_{1}$), indicating that diseased individuals die and experience both events, a transition that is not explicitly considered in copula‐based models.

Recently proposed models motivated by the IDM methodology suggest fitting a Cox proportional hazards (PH) model with shared frailty for each transition [3, 9, 12]. These approaches rely on modeling the hazard function while differentiating between two types of hazards: a hazard function, conditioned on the unobserved frailty variate and the observed time‐independent covariates, and a marginal hazard, defined after integrating out the frailty and depending only on the covariates. However, results are interpreted on the level of hazards and usually communicated as hazard ratios that might be misinterpreted as a relative risk. Sutradhar and Austin [14] emphasize that hazard ratios can only be used to explain the direction of the relative risk rather than the magnitude, and therefore their interpretation may be misleading if not carried out correctly. A further limitation arises due to the non‐collapsibility of the hazard ratios [15, 16], implying that the magnitude of the effect measure is not the same when conditioning on different sets of covariates. Consequently, the conditional hazard ratio differs from the marginal hazard ratio. Lastly, applying the Cox PH model relies on the assumption of a time constant hazard ratio. If the PH assumption is violated, the parameters estimated by the Cox model depend on the study‐specific censoring distribution. This remains true even in the presence of independent censoring [16]. To overcome the described disadvantages an appealing alternative is to apply an accelerated failure time (AFT) model instead. The major advantage of this approach is its intuitive interpretation, as the effect of the covariates is observed directly on the time‐to‐event and not on the hazard scale.

In recent literature, two models have been using an AFT approach for semi‐competing risks data. Kats and Gorfine [7] introduce a gamma‐frailty AFT model, where the frailty variable acts multiplicatively on the hazards of the error terms. A semi‐parametric maximum likelihood is used to estimate the model parameters via an Expectation–Maximization (EM) algorithm. Lee et al. [4] propose an additive frailty AFT approach, implementing a parametric log‐normal model and a semi‐parametric model based on Dirichlet process mixtures. Comparing both approaches, only the model of Lee et al. adopts interval censoring for non‐terminal events. In terms of inference, a Bayesian framework has been suggested. However, the prior distribution specification in complex Bayesian models may require careful consideration and extensive sensitivity analysis, which can hamper the straightforward application of the model in practice.

This article seeks to introduce an alternative AFT model within a frequentist framework, employing the maximization of the likelihood function and accounting for the complex study design associated with semi‐competing risks data. We suggest a parametric approach and assume a Weibull distribution for the time‐to‐event variables. Further, the model is defined by using age as the timescale. Therefore the observed data are left truncated as the study participants were not observed since birth. Accordingly, we incorporate left truncation in the final likelihood function to account for a delayed entry, which means that only individuals who are healthy and have survived up to study entry are included in the study. Consequently, left truncation has to be considered for both age at disease onset and age of death. In addition, interval censoring for disease occurrence is assumed, as the exact age at disease onset is rarely observed. In cohort studies, the diagnosis is usually made on intermittent visits and therefore the disease onset is only known to be in the interval between the first visit with diagnosis and the previous one. Finally, we connect the three AFT models for the transitions of the IDM by adding a trivariate normally distributed random effect that enables to account for potential correlations between the different event ages.

The remainder of this article is organized as follows: in Section 2, we introduce the general framework of AFT models and explain its relation to the IDM. We derive the likelihood function by explicitly defining the single contribution terms depending on four possible observed scenarios. Thereby, we consider a Markovian‐type model. To assess the model accuracy, we conduct a simulation study, which is described in Section 3. The application of the PAQUID study data, investigating the influence of various risk factors on the occurrence of dementia, is described in detail in Section 4. We show that our parametric AFT model leads to plausible results, confirming the findings of existing methods on the same data. Finally, we summarize the overall findings and provide a general overview of the strengths and limitations of our model.

## 2 Methods: An AFT Model for Semi‐Competing Risks Data

In this section, we present our parametric AFT model for semi‐competing risks data in detail. We first briefly explain the general framework of AFT models, followed by a description of their application in the IDM context. As the underlying data are subject to a complex censoring mechanism, we provide a general overview by illustrating the four possibly observed settings and explaining their contribution to the likelihood function. We propose a Markovian‐type specification of the likelihood and describe its derivation.

### 2.1 Model Specification

AFT models differ from the PH model in that they do not focus on modeling the hazard function, but offer the possibility of examining the effects of covariates on the time‐to‐event variables directly. As the present model is defined on the age scale, we will refer to the ages at which events occur in the upcoming sections, rather than the times to an event.

To introduce the general definition of an AFT model, let $T_{1}$ and $T_{2}$ be the age at disease onset and age at death, respectively. Suppose further $X={\left(1,X_{1},\ldots ,X_{d-1}\right)}^{⊤}\in {\mathbb{R}}^{d}$ is a d‐dimensional vector of age‐independent covariates and an intercept. In an AFT model, the outcome is modeled on a logarithmic scale:

(1) $$\log\left(T_{1}\right)=X^{⊤}\beta +\kappa \epsilon ,$$

where $\beta \in {\mathbb{R}}^{d}$ is a vector of unknown regression coefficients, κ is a scale parameter and ϵ denotes an error term. A simple transformation of Equation (1) yields

$$T_{1}=\exp(X^{⊤}\beta +\kappa \epsilon )=\exp(\beta_{0})\exp(\beta_{1}X_{1})...\exp(\beta_{d-1}X_{d-1})\exp(\kappa \epsilon )$$

Here, the covariates have a direct multiplicative impact on age, which differs from the PH model where they affect the hazard function.

To fully describe the IDM as given in Figure 1, for each event and underlying transition an AFT model is specified:

(2) $$\begin{array}{l} \log\left(T_{1}\right)=X^{⊤}\beta_{i}+\kappa_{i}\varepsilon_{i}+u_{i},\;T_{1}>0,\\ \log\left(T_{2}\right)=X^{⊤}\beta_{m_{0}}+\kappa_{m_{0}}\varepsilon_{m_{0}}+u_{m_{0}},\;T_{2}>0, \end{array}$$

(3) $$\log\left(T_{2}\right)=X^{⊤}\beta_{m_{1}}+\kappa_{m_{1}}\varepsilon_{m_{1}}+u_{m_{1}},\;T_{2}>T_{1}>0,$$

where $\beta_{p}=\left(\beta_{0,p},\beta_{1,p},\ldots \beta_{d-1,p}\right)$ are transition‐specific regression coefficient vectors, $\kappa_{p}$ are scale parameters, $\varepsilon_{p}$ denote random error terms and $u_{p}$ are random effects, $p\in \left\{i,m_{0},m_{1}\right\}$, respectively. The incidence is described by modeling the age at disease onset $T_{1}$ and estimating the corresponding transition‐specific effects, whereas the two mortalities ($m_{0},m_{1}$) are defined through modeling $T_{2}$ depending on whether a study participant is diseased or not (Equations 2 and 3, respectively). The random effects are assumed to be multivariate normally distributed,

(4) $$u=\left(\begin{array}{c} u_{i}\\ u_{m_{0}}\\ u_{m_{1}} \end{array}\right)∼N\left[\left(\begin{array}{c} 0\\ 0\\ 0 \end{array}\right),\left(\begin{array}{ccc} s_{i}^{2} & c_{i,m_{0}} & c_{i,m_{1}}\\ & s_{m_{0}}^{2} & c_{m_{0},m_{1}}\\ & & s_{m_{1}}^{2} \end{array}\right)\right],$$

with expected value 0, transition‐specific variances $s_{p}^{2}$ and covariances $c_{i,m_{0}},c_{i,m_{1}},c_{m_{0},m_{1}}$.

Finally, $T_{1},T_{2},T_{2}|T_{1}$ follow a particular distribution $F_{p}$, $p\in \left\{i,m_{0},m_{1}\right\}$, for which different assumptions such as Gompertz, Weibull, Extreme value, Log‐normal or Log‐logistic are possible.

### 2.2 Observed Data

**FIGURE 2:**
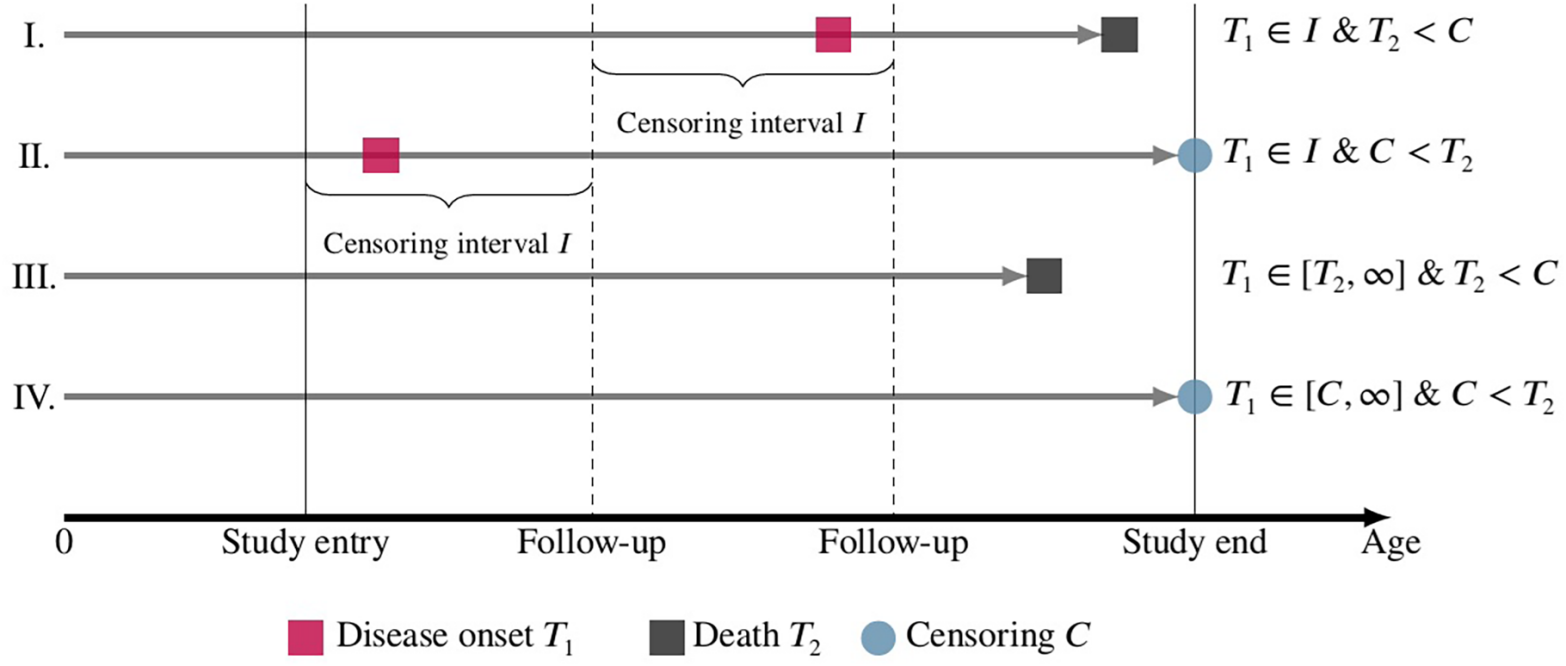
Four observed cases for semi‐competing risks data.

The age‐at‐event outcomes in semi‐competing risks settings are subject to complex censoring mechanisms. To illustrate the four possible cases observed within the described setting as illustrated in Figure 2 and to introduce the corresponding notation for the likelihood function, we consider a study of j=1,…,n participants and let $T_{1j}$ and $T_{2j}$ be the age at disease onset and age at death for the *j*th individual, respectively. As it is assumed for the IDM that each individual starts in the initial state “healthy,” only incident cases are observed during the study period. To account for the potential resulting immortal time bias, we assume that the distributions of age at disease onset and age at death are left truncated at the age at study entry, indicated by $L_{j}$ and hence require that $L_{j}<\min\left(T_{1j},T_{2j}\right)$. We further assume that $T_{2j}$ is subject to right censoring with age at censoring denoted by $C_{j}$. Hence, the last observed age for a study participant is $Y_{2j}=\min\left(T_{2j},C_{j}\right)$ with corresponding indicator function for the mortality $\delta_{2j}=1\left(T_{2j}<C_{j}\right)$ that describes whether an observation is censored ($\delta_{2j}=0$) or not ($\delta_{2j}=1$).

In cohort studies, the exact age at disease onset is often unknown, since diseases are usually diagnosed at follow‐up visits. Therefore, $T_{1j}$ is subject to interval censoring. The lower and upper bounds of the interval are given by the last known age without diagnosed disease and the first observed age after disease onset. We denote the corresponding interval by $I_{j}=\left[T_{\mathrm{lj}},T_{\mathrm{rj}}\right]$ and the disease diagnosis indicator by $\delta_{1j}=1\left(T_{\mathrm{rj}}<\min\left(T_{2j},C_{j}\right)\right)$. In the cases where death occurs first or the study terminates before diagnosis, that is, $\delta_{1j}=0$, the censoring interval is $I_{j}=\left[T_{\mathrm{lj}},T_{\mathrm{rj}}\right]=\left[Y_{2j},\infty \right)$. Hence, the actual observed set of variables for the *j*th study participant is given by $O_{j}=\left(I_{j},\delta_{1j},Y_{2j},\delta_{2j},L_{j},X_{j}\right)$.

### 2.3 Model Estimation

The likelihood for the model is derived in several steps. We first define the joint density of $\left(T_{1},T_{2}\right)$(Step 1), then incorporate left truncation (Step 2) and finally interval censoring and right censoring (Step 3).

*Step 1: Definition of joint density of* ($T_{1},T_{2}$)

Without considering left truncation, that is, individuals are observed since birth (L=0) and there is no right or interval censoring, the joint density of $\left(T_{1},T_{2}\right)$ denoted as $g_{T_{1},T_{2}|X}$, is given by [17]:

(5) $$\begin{array}{cc} g_{T_{1},T_{2}|X,}\left(t_{1},t_{2}|x\right) & ={\left\{\lambda_{i}\left(t_{1}|x\right)\right\}}^{\delta_{1}}S_{i}\left(t|x\right){\left\{\lambda_{m_{0}}\left(t_{2}|x\right)\right\}}^{\left(1-\delta_{1}\right)}\\ & \;S_{m_{0}}\left(t|x\right){\left\{\lambda_{m_{1}}\left(t_{2}|t_{1},x\right)S_{m_{1}}(t_{2}|t_{1},x)\right\}}^{\delta_{1}}, \end{array}$$

with $\lambda_{p}\left(.\right)$ and $S_{p}\left(.\right)$ being the hazard and survival function for the respective transition $p\in \left\{i,m_{0},m_{1}\right\}$ and $t=\min\left(t_{1},t_{2}\right)$. For model definition, we use the Markovian property [18], thereby assuming that the probability of transitioning to a new state depends only on the current state occupied. In particular, for $m_{1}$ this implies, that the corresponding distribution of $T_{2}|T_{1}$ does not rely on any previous state of the model. Instead, it is exclusively defined within the interval from disease onset to death:

$$S_{m_{1}}\left(t_{2}|t_{1},x\right)=\exp\left\{-\int_{t_{1}}^{t_{2}}\lambda_{m_{1}}\left(u|x\right)\mathrm{du}\right\}=\frac{S_{m_{1}}\left(t_{2}|x\right)}{S_{m_{1}}\left(t_{1}|x\right)}.$$

*Step 2: Left truncation for* $\left(T_{1},T_{2}\right)$

Next, we incorporate left truncation into the joint density of $\left(T_{1},T_{2}\right)$ and hence define the conditional probability [12, 17]:

(6) $$f_{T_{1},T_{2},L|X}\left(t_{1},t_{2},l|x\right)=\frac{g_{T_{1},T_{2}|X}\left(t_{1},t_{2}|x\right)g_{L}\left(l\right)}{P\left(L<\min\left(T_{1},T_{2}\right)|X\right)}1\left(l<\min\left(t_{1},t_{2}\right)\right),$$

where $g_{L}\left(.\right)$ denotes the probability density function (pdf) of L. The truncation probability in the denominator can be expressed as follows [12, 17]:

$$\begin{array}{ll} P\left(L<\min\left(T_{1},T_{2}\right)|X\right) & =P\left(T_{1}>L\cap T_{2}>L|X\right),\\ & \;=P\left(T_{1}>L,T_{2}>L|X\right),\\ & \;=\int_{l}^{\infty }\int_{l}^{\infty }\int_{0}^{\infty }f_{i}\left(t_{1}|x\right)f_{m_{0}}\left(t_{2}|x\right)g_{L}\left(l\right)dldt_{1}dt_{2},\\ & \;=\int_{0}^{\infty }g_{L}\left(l\right)S_{i}\left(l|x\right)S_{m_{0}}\left(l|x\right)dl. \end{array}$$

Inserting into Equation (6) and multiplying both denominator and numerator by $S_{i}\left(l|x\right)S_{m_{0}}\left(l|x\right)$ we obtain:

(7) $$\begin{array}{cc} f_{T_{1},T_{2},L|X}\left(t_{1},t_{2},l|x\right) & =\frac{g_{T_{1},T_{2}|X}\left(t_{1},t_{2},l|x\right)1\left(l<\min\left(t_{1},t_{2}\right)\right)}{S_{i}\left(l|x\right)S_{m_{0}}\left(l|x\right)}\\ & \;\times \frac{g_{L}\left(l\right)S_{i}\left(l|x\right)S_{m_{0}}\left(l|x\right)}{\int_{0}^{\infty }g_{L}\left(l\right)S_{i}\left(l|x\right)S_{m_{0}}\left(l|x\right)\mathrm{dl}}, \end{array}$$

That is, the product of the conditional pdf of $T_{1},T_{2}$ given L and the marginal pdf of L. Without any parametric assumptions on $g_{L}\left(.\right)$, the maximum of $f_{T_{1},T_{2},L|X}\left(t_{1},t_{2},l|x\right)$ can be obtained by considering only the first term of the product in Equation (7) [12, 17, 19].

Hence, the joint density of $\left(T_{1},T_{2},L\right)$, without interval censoring for $T_{1}$ and right censoring for $T_{2}$ is given by:

(8) $$\begin{array}{cc} & f_{T_{1},T_{2},L|X}\left(t_{1},t_{2},l|x\right)\\ & \;=\frac{{\left\{\lambda_{i}\left(t_{1}|x\right)\right\}}^{\delta_{1}}S_{i}\left(t|x\right){\left\{\lambda_{m_{0}}\left(t_{2}|x\right)\right\}}^{\left(1-\delta_{1}\right)}S_{m_{0}}\left(t|x\right){\left\{\lambda_{m_{1}}\left(t_{2}|x\right)S_{m_{1}}(t_{2}|x)\right\}}^{\delta_{1}}}{S_{i}\left(l|x\right)S_{m_{0}}\left(l|x\right)S_{m_{1}}{\left(t_{1}|x\right)}^{\delta_{1}}}. \end{array}$$

*Step 3: Interval censoring for* $T_{1}$ *and right censoring for* $T_{2}$

So far, we considered the age at disease onset as exactly known. However, in practice, we often only know the interval in which the true age at disease onset lies. To incorporate the respective interval censoring in the joint density, the term $\lambda_{i}{\left(t_{1}|x\right)}^{\delta_{1}}S_{i}\left(t|x\right)$ from Equation (8), is substituted with the difference of the survival functions evaluated at the interval boundaries $\left(S_{i}\left(t_{l}|x\right)-S_{i}\left(t_{r}|x\right)\right)$. Thereby, we express the probability of observing the age at disease onset in the interval between the first visit with a diagnosed disease and the last one without diagnosis $\left[T_{l},T_{r}\right]$.

We further considered the distribution of $T_{2}|T_{1}$ (describing $m_{1}$) as left‐truncated by the disease onset $T_{1}$. As age at disease onset is usually unknown, there are two options for truncation of $T_{2}|T_{1}$: (1) at the right boundary of the censoring interval $T_{r}$, that is, the age at the follow‐up visit when the diagnosis is reported for a first time, or (2) at the midpoint of the censoring interval $T_{m}=\left(T_{r}+T_{l}\right)/2$. In some studies, the follow‐up visits might be extended to longer intervals, leading to a larger bias if the first option is chosen. Therefore, we selected the second option.

In the final step, we incorporate right censoring of $T_{2}$ by including the last observed age in the study, $Y_{2}=\min\left(T_{2},C\right)$, in the final likelihood. For a better overview, the likelihood is decomposed into the four contributions as shown in Table 2, where we condition on the observed data **X** and on the unobserved random effects vector **u**. The final parameter vector of the model is $\theta =\left(\beta_{i}^{⊤},\beta_{m_{0}}^{⊤},\beta_{m_{1}}^{⊤},\kappa_{i},\kappa_{m_{0}},\kappa_{m_{1}},s_{i}^{2},s_{m_{0}}^{2},s_{m_{1}}^{2},c_{i,m_{0}},c_{i,m_{1}},c_{m_{0},m_{1}}\right)$. Inference for θ is based on the marginal likelihood function ℒ(θ), that is, the conditional likelihood integrated over the random effects vector:

(9) $$\begin{array}{l} ℒ\left(\theta \right)=\prod_{j=1}^{n}\int {ℒ}_{1}{\left(t_{\mathrm{rj}},t_{\mathrm{lj}},y_{2j}|x_{j},u_{j}\right)}^{\delta_{1j}\delta_{2j}}{ℒ}_{2}{\left(t_{\mathrm{rj}},t_{\mathrm{lj}},y_{2j}|x_{j},u_{j}\right)}^{\delta_{1j}\left(1-\delta_{2j}\right)}\\ \;{ℒ}_{3}{\left(t_{\mathrm{rj}},t_{\mathrm{lj}},y_{2j}|x_{j},u_{j}\right)}^{\delta_{2j}\left(1-\delta_{1j}\right)}{ℒ}_{4}{\left(t_{\mathrm{rj}},t_{\mathrm{lj}},y_{2j}|x_{j},u_{j}\right)}^{\left(1-\delta_{1j}\right)\left(1-\delta_{2j}\right)}f\left(u_{j}\right)du_{j} \end{array}$$

with ${ℒ}_{i},i=1,\ldots ,4$ as given in Table 2 and f(u) being the probability density function of the random effects vector. Usually, −log(ℒ(θ)) can be exactly numerically minimized over θ to estimate the parameter vector. However, the integral in (9) has no closed form, and therefore we apply Gaussian quadrature approximation which numerically estimates the integral through a weighted summation across predetermined abscissas for the random effects [20]. A good choice of quadrature points is essential for proper estimation. To fit the model, we apply SAS PROC NLMIXED with non‐adaptive Gaussian quadrature, but in general, any procedure that allows for the estimation of a self‐written likelihood function and incorporation of multivariate random effects could be used.

**TABLE 2:** The four possible contributions to the likelihood, depending on the disease diagnosis and death status.

Case	Description	$\delta_{1}$	$\delta_{2}$	Likelihood contribution
0	Death or disease onset occurs before study entry.	0	0	—
I.	Disease onset between two follow‐up visits and death occurs before the study ends.	1	1	${ℒ}_{1}=\frac{\left(S_{i}\left(t_{l}|x,u\right)-S_{i}\left(t_{r}|x,u\right)\right)S_{m_{0}}\left(t_{r}|x,u\right)f_{m_{1}}\left(y_{2}|x,u\right)}{S_{i}\left(l|x,u\right)S_{m_{0}}\left(l|x,u\right)S_{m_{1}}\left(t_{m}|x,u\right)}$
II.	Disease onset between two follow‐up visits and death is right censored at the study end.	1	0	${ℒ}_{2}=\frac{\left(S_{i}\left(t_{l}|x,u\right)-S_{i}\left(t_{r}|x,u\right)\right)S_{m_{0}}\left(t_{r}|x,u\right)S_{m_{1}}\left(y_{2}|x,u\right)}{S_{i}\left(l|x,u\right)S_{m_{0}}\left(l|x,u\right)S_{m_{1}}\left(t_{m}|x,u\right)}$
III.	No disease diagnosis at last follow‐up visit and death occurs before the study ends.	0	1	${ℒ}_{3}=\frac{S_{i}\left(y_{2}|x,u\right)f_{m_{0}}\left(y_{2}|x,u\right)}{S_{i}\left(l|x,u\right)S_{m_{0}}\left(l|x,u\right)}$
IV.	No disease diagnosis at last follow‐up visit and death is right censored at the study end.	0	0	${ℒ}_{4}=\frac{S_{i}\left(y_{2}|x,u\right)S_{m_{0}}\left(y_{2}|x,u\right)}{S_{i}\left(l|x,u\right)S_{m_{0}}\left(l|x,u\right)}$

## 3 Simulation Study

To assess the performance of our model, we conducted a simulation study. The corresponding source code was written in SAS 9.4 [21] and can be found in the Zenodo repository https://zenodo.org/records/17652004.

For the simulation study, we abstain from comparing the model's performance with existing competing methods at this first stage of the method development as suggested by Heinze et al. [22].

### 3.1 Simulation Scenarios

We simulated 12 scenarios with 1000 studies each and varied the age at censoring c, the initial sample size n, and the mean age at death for diagnosed individuals $E_{m_{1}}$ (Table 3). The age at censoring is drawn from a uniform distribution 𝒰 considering two options: early and late censoring assuming 𝒰(85,95) and 𝒰(95,105), respectively. Further, we simulated three different initial study sizes with n=1200, n=2400, and n=500 participants. Note that the size of the final cohort for analysis is influenced by the proportion of generated age‐at‐event observations that exceed the age at study entry. Next, the age‐at‐event observations are derived from transition‐specific Weibull distributions. We considered two possible distributions for the age at death following disease diagnosis with expected values $E_{m_{1}}=65$ or $E_{m_{1}}=80$, respectively. The first one is motivated by the PAQUID study which documented a substantial effect of dementia on life expectancy (see Section 4), whereas the second scenario reflects a disease with a less drastic effect on the life expectancy after diagnosis. To define the corresponding Weibull distributions given by $W\left(\exp\left(\beta_{0,m_{1}}+\beta_{1,m_{1}}X_{1}+u_{m_{1}}\right),\kappa_{m_{1}}\right)$, we used the estimated parameters from the PAQUID study and keep all but the intercept $\beta_{0,m_{1}}$ fixed that is varied depending on the expected value. For the age at disease onset and the age at death for individuals without diagnosis, we used the same approach with expected values $E_{i}=95$ and $E_{m_{0}}=85$, respectively. Additionally, the age at which participants enter the study is drawn from 𝒰(55,60).

**TABLE 3:** Overview of the simulation study settings and the varied factors.

Setting	Varying parameters		
	Age at censoring	$E_{m_{1}}$	Initial sample size n
E65 L	𝒰(85,95)	65	2400
E80 L	𝒰(85,95)	80	2400
L65 L	𝒰(95,105)	65	2400
L80 L	𝒰(95,105)	80	2400
E65 M	𝒰(85,95)	65	1200
E80 M	𝒰(85,95)	80	1200
L65 M	𝒰(95,105)	65	1200
L80 M	𝒰(95,105)	80	1200
E65 S	𝒰(85,95)	65	500
E80 S	𝒰(85,95)	80	500
L65 S	𝒰(95,105)	65	500
L80 S	𝒰(95,105)	80	500

Note that the censoring and truncation probability are not fixed, but depend on the generated age at study entry and the age at censoring. Therefore, scenarios with early censoring such as E65 and E80 represent settings with larger proportions of censored observations in each transition. For an overview of the final sample size and the number of events per transition, we refer to Table A1: Supporting Information and for the respective censoring proportions to Table A2: Supporting Information.

### 3.2 Data Generation

Data generation was conducted following partly the approach of Lee et al. [12] using the following steps:

1. Simulate n random effects for each transition $u_{i}$, $u_{m_{0}}$ and $u_{m_{1}}$ from a trivariate normal distribution:
$$\left(\begin{array}{c} u_{i}\\ u_{m_{0}}\\ u_{m_{1}} \end{array}\right)∼N\left[\left(\begin{array}{c} 0\\ 0\\ 0 \end{array}\right),\left(\begin{array}{ccc} \sigma_{i}^{2} & 0 & c_{i,m_{1}}\\ & \sigma_{m_{0}}^{2} & 0\\ & & \sigma_{m_{1}}^{2} \end{array}\right)\right].$$
  The transition‐specific variances are set at $\sigma_{i}^{2}=0.008237$, $\sigma_{m_{0}}^{2}=0.000714$, $\sigma_{m_{1}}^{2}=0.000063$, and $c_{i,m_{1}}=0.000115$. Parameters are chosen based on findings from the PAQUID study (Section 4).
2. Generate observations of a binary random variable indicating the sex (1 = male, 0 = female) of each individual. The observations x are drawn from a Bernoulli distribution ($X_{1}∼\mathrm{Ber}\left(p\right)$) with p=0.4 motivated by the PAQUID study (Section 4).
3. Generate the age at disease onset $t_{1}$ and the age at death for disease‐free individuals $t_{2}$ for n study participants from the two predefined Weibull distributions: $W\left(\exp\left(X^{⊤}\beta_{i}+u_{i}\right),\kappa_{i}\right)$ and $W\left(\exp\left(X^{⊤}\beta_{m_{0}}+u_{m_{0}}\right),\kappa_{m_{0}}\right)$.
4. If $t_{1}>t_{2}$, that is, the generated age at disease onset is greater than the age at death, only the latter is observed for the respective participant.
5. In case $t_{1}<t_{2}$ both events are observable. Then, the age at death following a diagnosis, $t_{2}|t_{1}$, is simulated using the third Weibull distribution: $W\left(\exp\left(X^{⊤}\beta_{m_{1}}+u_{m_{1}}\right),\kappa_{m_{1}}\right)$.
6. For generating $t_{2}|t_{1}$, we apply the inversion method as proposed by Beyersmann et al. [23]. It is suggested to use the direct relation between the cumulative density function F(.) and the cumulative hazard function Λ(.) given by F(t)≔P(T≤t)=1−exp(−Λ(t)), and hence to simulate the age at death after diagnosis by applying
$$\begin{array}{cc} & T_{2}|T_{1}=F_{m_{1}}^{-1}\left(U\right)=\Lambda_{m_{1}}^{-1}\left(-\log\left(1-U\right)\right),\text{where}\;U∼𝒰\left(0,1\right)\;\\ & \;\text{with}\;𝒰\;\text{being}\;\text{the}\;\text{uniform}\;\text{distribution}\;\mathrm{on}\;\left[0,1\right]. \end{array}$$
  For generating data such that $t_{2}|t_{1}>t_{1}$, the cumulative hazard function is truncated at the age of the disease onset, $\Lambda_{m_{1}}\left(t\right)=\int_{t_{1}}^{t}\lambda_{m_{1}}\left(u\right)\mathrm{du}$, with $\lambda_{m_{1}}$ being the hazard function of the respective conditional Weibull distribution of $T_{2}|T_{1}$.
7. Generate age at study entry L ∼𝒰(.,.) and age at censoring C∼𝒰(.,.) for each participant from respective uniform distributions.
8. Depending on the generated age at baseline l, age at censoring c and age at both events, assign the respective status $\delta_{1}=1$ for a diagnosed individual and $\delta_{2}=1$ for an observed death. For example, if $l<t_{1}<c$ and $c<t_{2}|t_{1}$ the given individual has entered the study, has been diagnosed during the follow‐up period ($\delta_{1}=1$), and censored before death ($\delta_{2}=0$).
9. Delete observations for which the age at study entry l is greater than the generated ages at event $t_{1}$ or $t_{2}$ as $L<\min\left(T_{1},T_{2}\right)$ is required (Section 2.3).
10. For diagnosed individuals, simulate the age at the visit at which the diagnosis is reported for the first time as well as the age at the previous visit, that is, the boundaries for the censoring interval $\left[t_{l},t_{r}\right]$. We assume that follow‐up visits take place at time intervals not longer than 6 years. Therefore the lower and upper bound of the respective interval are defined by generating two uniformly distributed random variables on the interval [0,3] and subtracting and adding them to the true age at disease onset $t_{1}$: $t_{r}=t_{1}+U$ and $t_{l}=t_{1}-U$ with U∼𝒰(0,3). Hence, we obtain an interval of randomly generated upper and lower bounds around the age of the disease onset. At last, the midpoint of the censoring interval is computed.

The final simulated data set contains the following observations for n individuals: age at baseline l, age at death $t_{2}$, left boundary for the age at disease onset $t_{l}$, right boundary for the age at disease onset $t_{r}$, midpoint of the censoring interval $t_{m}$ and a binary covariate indicating the sex of the individual.

### 3.3 Estimation Methods

We fitted two different models with respect to the variance–covariance structure of the random effects: model HomVar, for which the random effects are not transition‐specific with $u∼N\left(0,s^{2}\right)$ being added to each linear predictor, assuming a homogenous variance and the model HetVar for which the variance–covariance matrix of the random effects is given by

(10) $$\left(\begin{array}{c} u_{i}\\ u_{m_{0}}\\ u_{m_{1}} \end{array}\right)∼N\left[\left(\begin{array}{c} 0\\ 0\\ 0 \end{array}\right),\left(\begin{array}{ccc} s_{i}^{2} & 0 & c_{i,m_{1}}\\ & s_{m_{0}}^{2} & c_{m_{0},m_{1}}\\ & & s_{m_{1}}^{2} \end{array}\right)\right].$$

We considered heterogeneous random effects variances for each transition, alongside a covariance term for age at disease onset and age at death following a diagnosis and a covariance term accounting for the dependency between the two mortalities. Further, an independence is assumed between disease onset and mortality without the disease ($c_{i,m_{0}}=0$), since the occurrence of death in this case is not associated with the disease.

The models are estimated using PROC NLMIXED in SAS, which facilitates the estimation of nonlinear mixed models by maximizing an approximation of the likelihood function integrated over the random effects. Among various options for integral approximation, we opted for a Gaussian quadrature with two quadrature points and a non‐adaptive approach. As an optimization technique, the double‐dogleg optimization (DBLDOG) was been applied. To determine starting values for optimization, we fit three univariate models without random effects, where the parameters for each transition ($i,m_{0},m_{1}$) are estimated and later employed as initial values for the final trivariate model.

### 3.4 Results

In reporting our results, we confine ourselves to the transition‐specific parameters: the Weibull shape parameter $\kappa_{p}$ and the regression coefficients $\left(\beta_{0,p},\beta_{1,p}\right)$ Additionally, to evaluate the precision of the estimated overall age at events, the mean and median of the three estimated Weibull distributions are examined: $E_{p}=\exp\left(\beta_{0,p}+X_{1}\beta_{1,p}\right)\Gamma \left(1+\frac{1}{\kappa_{p}}\right)$ and ${\mathrm{Med}}_{p}=\exp\left(\beta_{0,p}+X_{1}\beta_{1,p}\right){\left(\log\left(2\right)\right)}^{1/\kappa_{p}}$, where Γ(.) denotes the gamma function. The performance of the model is measured by median bias, mean squared error (MSE), the number of converged simulation runs, and empirical coverage to the 95% level, computed based on Wald‐type confidence intervals.

The results concerning bias are given in Tables 4 and 5 (model parameters) and Tables A6 and A8: Supporting Information (Weibull mean and median) in the Supporting Information for HomVar and HetVar, respectively.

**TABLE 4:** Model HomVar. Median bias and mean squared error (MSE) over 1000 iterations for each simulations scenario: E65, E80, L65, L80, and each initial sample size *n* = 2400, *n* = 1200, and *n* = 500.

Setting	Parameter	*n* = 2400		*n* = 1200		*n* = 500	
		Median bias	MSE	Median bias	MSE	Median bias	MSE
E65	$b_{0,m_{1}}$	0.01	0.00	0.01	0.00	0.02	0.00
E65	$b_{0,m_{0}}$	0.00	0.00	0.00	0.00	0.00	0.00
E65	$b_{0,i}$	0.01	0.00	0.00	0.00	0.01	0.00
E65	$b_{1,m_{1}}$	−0.01	0.00	−0.01	0.00	−0.01	0.00
E65	$b_{1,m_{0}}$	0.00	0.00	0.00	0.00	0.00	0.00
E65	$b_{1,i}$	0.00	0.00	0.00	0.00	0.00	0.00
E65	$\kappa_{m_{1}}$	0.95	1.38	0.93	1.78	0.94	3.13
E65	$\kappa_{m_{0}}$	0.25	0.24	0.23	0.43	0.20	1.00
E65	$\kappa_{i}$	1.09	1.44	1.11	1.71	1.06	2.33
E80	$b_{0,m_{1}}$	0.00	0.00	0.00	0.00	0.00	0.00
E80	$b_{0,m_{0}}$	0.00	0.00	0.00	0.00	0.00	0.00
E80	$b_{0,i}$	0.01	0.00	0.01	0.00	0.01	0.00
E80	$b_{1,m_{1}}$	0.00	0.00	0.00	0.00	0.00	0.00
E80	$b_{1,m_{0}}$	0.00	0.00	0.00	0.00	0.00	0.00
E80	$b_{1,i}$	0.00	0.00	0.00	0.00	0.00	0.00
E80	$\kappa_{m_{1}}$	0.14	1.28	0.07	2.67	−0.04	7.60
E80	$\kappa_{m_{0}}$	0.32	0.29	0.30	0.49	0.23	1.22
E80	$\kappa_{i}$	0.92	1.09	0.88	1.28	0.80	1.93
L65	$b_{0,m_{1}}$	0.03	0.00	0.03	0.00	0.03	0.00
L65	$b_{0,m_{0}}$	0.00	0.00	0.00	0.00	0.00	0.00
L65	$b_{0,i}$	0.00	0.00	0.00	0.00	0.00	0.00
L65	$b_{1,m_{1}}$	−0.01	0.00	−0.01	0.00	−0.01	0.00
L65	$b_{1,m_{0}}$	0.00	0.00	0.00	0.00	0.00	0.00
L65	$b_{1,i}$	0.00	0.00	0.00	0.00	0.00	0.00
L65	$\kappa_{m_{1}}$	1.42	2.21	1.36	2.38	1.35	3.00
L65	$\kappa_{m_{0}}$	0.35	0.24	0.32	0.33	0.31	0.68
L65	$\kappa_{i}$	1.33	1.89	1.32	2.05	1.25	2.35
L80	$b_{0,m_{0}}$	−0.01	0.00	−0.01	0.00	0.00	0.00
L80	$b_{0,i}$	0.00	0.00	0.00	0.00	0.00	0.00
L80	$b_{1,m_{1}}$	0.00	0.00	0.00	0.00	0.00	0.00
L80	$b_{1,m_{0}}$	0.00	0.00	0.00	0.00	0.00	0.00
L80	$b_{1,i}$	0.00	0.00	0.00	0.00	0.00	0.00
L80	$\kappa_{m_{1}}$	0.03	0.54	−0.05	1.33	−0.19	3.75
L80	$\kappa_{m_{0}}$	0.46	0.33	0.42	0.47	0.30	0.88
L80	$\kappa_{i}$	1.16	1.50	1.13	1.59	1.09	2.04

**TABLE 5:** Model HetVar. Median bias and mean squared error (MSE) over 1000 iterations for each simulations scenario: E65, E80, L65, L80, and each initial sample size *n* = 2400, *n* = 1200, and *n* = 500.

Setting	Parameter	*n* = 2400		*n* = 1200		*n* = 500	
	Median bias	MSE	Median bias	MSE	Median bias	MSE
E65	$b_{0,m_{1}}$	0.01	0.00	0.01	0.00	0.02	0.00
E65	$b_{0,m_{0}}$	0.00	0.00	0.00	0.00	0.00	0.00
E65	$b_{0,i}$	0.01	0.00	0.00	0.00	0.01	0.00
E65	$b_{1,m_{1}}$	−0.01	0.00	−0.01	0.00	−0.01	0.00
E65	$b_{1,m_{0}}$	0.00	0.00	0.00	0.00	0.00	0.00
E65	$b_{1,i}$	0.00	0.00	0.00	0.00	0.00	0.00
E65	$\kappa_{m_{1}}$	0.95	1.36	0.94	1.74	0.94	3.02
E65	$\kappa_{m_{0}}$	0.13	0.17	0.12	0.32	0.09	0.78
E65	$\kappa_{i}$	1.09	1.42	1.11	1.68	1.06	2.20
E65	$s_{i}^{2}$	0.01	—	0.01	—	0.01	—
E65	$s_{m_{0}}^{2}$	0.00	—	0.00	—	0.00	—
E65	$s_{m_{1}}^{2}$	0.00	—	0.00	—	0.00	—
E65	$c_{i,m_{1}}$	−4.00	—	−4.00	—	−4.00	—
E65	$c_{m_{0},m_{1}}$	0.00	—	0.00	—	0.00	—
E80	$b_{0,m_{1}}$	0.00	0.00	0.00	0.00	0.00	0.00
E80	$b_{0,m_{0}}$	0.00	0.00	0.00	0.00	0.00	0.00
E80	$b_{0,i}$	0.01	0.00	0.01	0.00	0.01	0.00
E80	$b_{1,m_{1}}$	0.00	0.00	0.00	0.00	0.00	0.00
E80	$b_{1,m_{0}}$	0.00	0.00	0.00	0.00	0.00	0.00
E80	$b_{1,i}$	0.00	0.00	0.00	0.00	0.00	0.00
E80	$\kappa_{m_{1}}$	0.14	1.20	0.07	2.44	0.06	6.00
E80	$\kappa_{m_{0}}$	0.16	0.20	0.15	0.36	0.11	0.80
E80	$\kappa_{i}$	0.92	1.07	0.89	1.25	0.83	1.88
E80	$s_{i}^{2}$	0.01	—	0.01	—	0.01	—
E80	$s_{m_{0}}^{2}$	0.00	—	0.00	—	0.00	—
E80	$s_{m_{1}}^{2}$	0.00	—	0.00	—	0.00	—
E80	$c_{i,m_{1}}$	−4.00	—	−4.00	—	−4.00	—
E80	$c_{m_{0},m_{1}}$	0.00	—	0.00	—	0.00	—
L65	$b_{0,m_{1}}$	0.03	0.00	0.03	0.00	0.03	0.00
L65	$b_{0,m_{0}}$	0.00	0.00	0.00	0.00	0.00	0.00
L65	$b_{0,i}$	0.00	0.00	0.00	0.00	0.00	0.00
L65	$b_{1,m_{1}}$	−0.01	0.00	−0.01	0.00	−0.01	0.00
L65	$b_{1,m_{0}}$	0.00	0.00	0.00	0.00	0.00	0.00
L65	$b_{1,i}$	0.00	0.00	0.00	0.00	0.00	0.00
L65	$\kappa_{m_{1}}$	1.42	2.19	1.37	2.35	1.36	2.97
L65	$\kappa_{m_{0}}$	0.22	0.15	0.19	0.23	0.19	0.49
L65	$\kappa_{i}$	1.33	1.88	1.32	2.03	1.26	2.29
L65	$s_{i}^{2}$	0.01	—	0.01	—	0.01	—
L65	$s_{m_{0}}^{2}$	0.00	—	0.00	—	0.00	—
L65	$s_{m_{1}}^{2}$	0.00	—	0.00	—	0.00	—
L65	$c_{i,m_{1}}$	−4.00	—	−4.00	—	−4.00	—
L65	$c_{m_{0},m_{1}}$	0.00	—	0.00	—	0.00	—
L80	$b_{0,m_{1}}$	0.00	0.00	0.00	0.00	0.00	0.00
L80	$b_{0,m_{0}}$	0.00	0.00	0.00	0.00	0.00	0.00
L80	$b_{0,i}$	0.00	0.00	0.00	0.00	0.00	0.00
L80	$b_{1,m_{1}}$	0.00	0.00	0.00	0.00	0.00	0.00
L80	$b_{1,m_{0}}$	0.00	0.00	0.00	0.00	0.00	0.00
L80	$b_{1,i}$	0.00	0.00	0.00	0.00	0.00	0.00
L80	$\kappa_{m_{1}}$	0.08	0.43	0.06	0.91	0.03	2.21
L80	$\kappa_{m_{0}}$	0.28	0.18	0.28	0.28	0.22	0.49
L80	$\kappa_{i}$	1.18	1.54	1.15	1.64	1.19	2.07
L80	$s_{i}^{2}$	0.01	—	0.01	—	0.01	—
L80	$s_{m_{0}}^{2}$	0.00	—	0.00	—	0.00	—
L80	$s_{m_{1}}^{2}$	0.00	—	0.00	—	0.00	—
L80	$c_{i,m_{1}}$	−4.00	—	−4.00	—	−4.00	—
L80	$c_{m_{0},m_{1}}$	0.00	—	0.00	—	0.00	—

In general, our findings indicate that both models perform well with minor deviations from the true values. In addition, changes in sample size do not substantially affect median bias. The HetVar model appears to perform slightly better. When evaluating the results for the estimated model parameters (Tables 4 and 5), the highest median bias is observed for $\kappa_{m_{1}}$ with values of 1.42 in scenario L65L for both models, HomVar and HetVar. In general, the shape parameters κ in each transition show larger deviations from the true values compared to the regression coefficients. The transition‐specific regression coefficients are estimated quite precise with the overall highest median bias of 0.03 for $\beta_{0,m_{1}}$ in scenario L65L for the HomVar Model. Finally, in terms of the Weibull mean and median, we again observe quite good results, since the highest median bias does not exceed 2.52 for the mean age at death after a diagnosis $E_{m_{1}}$ of women and when applying the HomVar model. In most of the scenarios, that is, 72% and 75% for HomVar and HetVar respectively, the Weibull mean and median age at the event are estimated with a median bias of less than a year, indicating a satisfying accuracy.

In terms of the mean squared error (MSE), we observe overall satisfactory results for all settings and the two applied models, as shown in Tables 4 and 5. Best results are observed for the regression parameters with all MSE values being less than 1, indicating low bias and low standard errors. Higher MSE values can be found for some shape parameters κ and some of the Weibull mean and median values, especially in the early censoring scenarios E65 and E80. This is caused by larger standard errors of the estimates, especially in transitions and scenarios with fewer events. Further, we observe an increase of the MSE values with a smaller sample size, which is due to larger standard errors of the estimates in those settings.

In terms of empirical coverage, we observe quite satisfactory results for the regression parameters $\beta_{0,p},\beta_{1,p},p\in \left\{i,m_{0},m_{1}\right\}$ as 85% of the estimates in the HomVar model and 87% of the estimates in the HetVar model demonstrate an empirical coverage greater than 90%. Similarly to median bias and MSE, we detect the worst results in assessing coverage of the Weibull shape parameters κ, especially in scenarios with a large initial sample size. The latter is not surprising as the standard error is getting larger with a smaller sample size. Therefore, we observe an increase in coverage when decreasing the sample size for the same scenario (see Tables A9–A12: Supporting Information). Once again, the results for HetVar model are slightly better compared to those of HomVar.

However, challenges arise when evaluating the results for the variance and covariance parameters of the HetVar model. Due to the increased number of estimated parameters and the complexity of the model, $s_{i}^{2},s_{m_{0}}^{2},s_{m_{1}}^{2},c_{i,m_{1}}$ and $c_{m_{0},m_{1}}$ remain at their initial values. Therefore, the median bias, MSE and coverage cannot be computed for these. Nevertheless, the results discussed so far demonstrate that the HetVar model should still be preferred to the simplified HomVar model, as it yields more accurate estimates in most cases. A direct comparison shows that 74% of the model parameters estimated by the HetVar model have a smaller median bias, and in 97% of cases, a smaller MSE, than the respective estimates of the HomVar model.

Finally, we examine the numerical robustness of our model and observe quite satisfactory results as convergence has been reached for each iteration, parameter, and scenario. We could not detect any problems also for smaller data sets with an initial sample size n=500 and a larger amount of censored observations as shown in Table A4: Supporting Information.

## 4 Real Data Application

For illustration purposes, we use data from the PAQUID (Personnes Agées Quid) research program [24], a longitudinal study designed to follow up a cohort of 3777 elderly individuals aged 65 years and older at baseline in 1988. Study participants were randomly sampled from communal electoral lists in the communities of two administrative areas of South‐Western France: Gironde and Dordogne. Three inclusion criteria had to be met: minimal age of 65 years by December 31, 1987, living at home at the time of the initial data collection, and a signed informed consent to participate in the study. The main aim was to study normal and pathological brain aging concentrating on non‐genetic risk factors for developing dementia. The study covers a broad spectrum of socio‐demographic variables about social support, living conditions and habits, subjective and objective health measures, personal medical history, current symptoms, and diseases. However, for our application, we consider a random sample of subjects from the original cohort with two additional explanatory, binary variables: sex and primary school diploma. The main event of interest is the age of dementia onset and death as a semi‐competing event. The respective subset of the original data set is freely accessible and can be found in the **R** [25] package SmoothHazard [26].

The analyzed cohort reports on 1000 study participants (422 males and 578 females), see Table 6. Among them, 762 individuals possess a primary school diploma.

**TABLE 6:** Descriptive overview of the random sample of the PAQUID study data provided in the SmoothHazard package: Age variables are given as mean and standard deviation (SD), and events are reported as frequencies and percentages.

	Female (*n* = 578)	Male (*n* = 422)	Total (*n* = 1000)
Number dementia cases (%)	126 (21.8%)	60 (14.2%)	186 (18.6%)
Number deaths (disease‐free) (%)	317 (54.8%)	280 (66.4%)	597 (59.7%)
Number deaths (after diagnosis) (%)	82 (14.2%)	45 (10.7%)	127 (12.7%)
Mean age at baseline (SD)	75.65 (7.05)	74.19 (6.45)	75.03 (6.84)
Mean age at dementia diagnosis (SD)	83.20 (7.32)	81.17 (6.84)	82.34 (7.2)
Mean age at death (disease‐free) (SD)	86.18 (6.89)	83.32 (6.87)	84.84 (7.01)
Mean age at death (after diagnosis) (SD)	88.82 (6.06)	85.29 (4.99)	87.58 (5.93)

At baseline, the mean age is 75.03, with slight differences observed between females and males. The mean age at dementia‐free death and death after dementia diagnosis are 84.84 and 87.58, respectively, without notable differences between females and males. The number of dementia diagnoses is low for both sexes, indicating a heavily censored data set. However, for the majority of the study population, death has been observed with 724 terminal events.

We fitted the two parametric AFT Markov models applied in the simulation study (HomVar and HetVar) and one without random effects to compare them based on AIC and BIC. To determine starting values for optimization, we suggest fitting three univariate models without random effects, where the parameters for each transition are estimated and subsequently used as initial values for the final trivariate model. In addition, a grid search algorithm has been applied to find proper initial values for the random effects. The results indicate a better fit of the HomVar model with a lower AIC = 6367.8 and BIC = 6431.6 compared to the respective values for the HetVar model (AIC = 6375.8, BIC = 6459.2) and to those of the model without random effects (AIC = 6417.0 and BIC = 6475.9).

Results in terms of parameter estimates, acceleration factors, and corresponding 95% confidence intervals are given in Table 7. As the Weibull AFT model allows for a simple transformation of the estimated parameters into hazard ratios, for the sake of completeness, we additionally provide them in Table 7. However, we emphasize and highly recommend reporting the acceleration factors for their better interpretability. A negative β indicates an acceleration of the age at the event, that is, experiencing disease onset or death earlier and in this matter a negative effect, whereas a positive sign points to deceleration of the age at the event and therefore demonstrates a positive effect.

**TABLE 7:** Estimated model parameters of the random sample of the PAQUID study with the HomVar model.

	Estimate β (95% confidence interval)	Acceleration factor exp(β) (95% confidence interval)	Hazard ratio (95% confidence interval)
Incidence i			
Intercept	4.65 (4.59, 4.70)	—	—
Sex (male)	0.03 (−0.02, 0.07)	1.03 (0.98, 1.07)	0.81 (0.56, 1.07)
Diploma (yes)	−0.05 (−0.11, 0.00)	0.95 (0.90, 0.99)	1.49 (0.92, 2.06)
Mortality $m_{0}$			
Intercept	4.50 (4.48, 4.52)	—	
Sex (male)	−0.04 (−0.06, −0.03)	0.96 (0.94, 0.97)	1.54 (1.29, 1.79)
Diploma (yes)	−0.001 (−0.02, 0.02)	1.00 (0.98, 1.02)	1.01 (0.82, 1.20)
Mortality $m_{1}$			
Intercept	4.45 (4.36, 4.54)	—	
Sex (male)	−0.08 (−0.13, −0.03)	0.92 (0.88, 0.97)	1.91 (1.16, 2.66)
Diploma (yes)	−0.02 (−0.09, 0.04)	0.98 (0.92, 1.03)	1.23 (0.62, 1.85)
$s^{2}$	0.000003 (−0.0001, 0.0001)	—	—

The results suggest a positive impact of being male on the age at dementia onset indicated by an acceleration factor of $\exp\left(\beta_{1,i}\right)=1.03$, that is, the age of dementia onset for a male is 1.03 times the respective age for a female.

The positive effect of being a male in terms of developing dementia is reversed with regard to the age at death. The respective acceleration factors indicate that the mean age at death for a male (with and without dementia) is 0.92 and 0.96 times the respective age for a female. Hence, females are at higher risk for developing dementia, whereas males die earlier compared to females, regardless of the dementia status.

Furthermore, the risk of getting a dementia diagnosis is higher for individuals with a primary school diploma. The respective acceleration factor indicates that the mean age at dementia onset in the educated group is $\exp\left(\beta_{2,i}\right)=0.95$ times the age in the group without a diploma. Individuals with dementia and a primary school diploma are also at higher risk for death ($\exp\left(\beta_{2,m_{1}}\right)=0.98$), whereas no relevant differences are observed for individuals without dementia ($\exp\left(\beta_{2,m_{0}}\right)=1.00$). Further, we estimated a variance of 0.0001, indicating that covariates account for much of the outcome variability. Our findings are in line with the results of other models applied to the same data set [27].

Based on the estimated distribution parameters, we are furthermore able to determine “patient profiles” that can be compared to each other. As depicted in Table 8, the expected age at dementia onset for a female without a primary school diploma is approximately 4 years lower than for a male without a diploma (97.90 vs. 100.63 years). On the other hand, the average age at death for a male is more than 3 years lower than for a female (82.18 vs. 85.70). Furthermore, for the age at death after dementia diagnosis, we again observe a lower value for males (73.87) in comparison to females (80.26).

**TABLE 8:** Estimated mean and median age at dementia onset, age of death, and age of death after diagnosis for different patient profiles.

	Mean (95% confidence interval)	Median (95% confidence interval)
Female without diploma		
Age at dementia onset	97.90 (92.85, 102.82)	99.31 (94.18, 104.31)
Age at death	85.70 (84.15, 87.58)	86.83 (85.27, 88.69)
Age at death (after diagnosis)	80.26 (74.15, 89.27)	81.41 (75.29, 90.44)
Male without diploma		
Age at dementia onset	100.63 (94.57, 106.30)	102.09 (95.91, 107.85)
Age at death	82.18 (80.65, 84.00)	83.26 (81.73, 85.05)
Age at death (after diagnosis)	73.87 (67.77, 82.23)	74.94 (68.81, 84.31)
Female with diploma		
Age at dementia onset	92.75 (90.41, 95.09)	94.08 (91.72, 96.46)
Age at death	85.60 (84.79, 87.22)	86.72 (85.55, 88.14)
Age at death (after diagnosis)	78.11 (70.38, 85.84)	79.24 (73.90, 87.85)
Male with diploma		
Age at dementia onset	95.33 (91.72, 98.72)	96.71 (93.03, 100.15)
Age at death	82.08 (80.78, 83.62)	83.16 (81.86, 84.66)
Age at death (after diagnosis)	71.89 (66.01, 81.38)	72.93 (67.04, 82.42)

**FIGURE 3:**
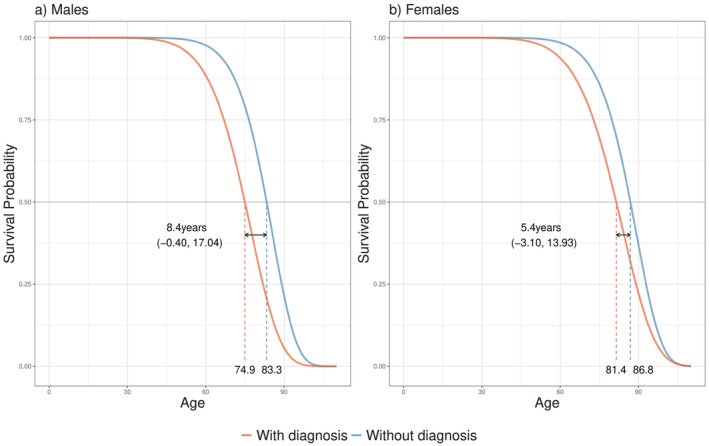
Estimated survival distribution of the age at death (with dementia and without dementia) for the males and females of the PAUIQD cohort and median survival difference with corresponding 95% CI.

However, the expected values of the estimated distributions might appear implausible compared to the descriptive results given in Table 6. At this point, we need to emphasize an important aspect regarding the interpretation of the results from our model. The AFT Markov model accounts for left truncation and right censoring. Hence, the expected age of dementia onset indicates what we would observe for a cohort that is studied from birth until all individuals die. A large mean does not imply a greater age at disease onset than the age at death, but simply the age at which dementia would manifest on average if the individual is still alive. On the other hand, the noticeably low mean and median age at death after diagnosis (between 71.89 and 81.41 depending on the profile) indicates that after disease onset, dementia largely affects life expectancy, resulting in a median difference of 8.4 years for males and 5.4 years for females compared to dementia‐free individuals (Figure 3). As a result, the application of our model provides more profound information on how dementia affects life expectancy across different patient profiles. In general, the inclusion of more covariates enables the definition of more diverse patient profiles and their comparison.

## 5 Discussion

In this article, we introduced a new parametric model for analyzing semi‐competing risks data for which a terminal event (e.g., death) might censor a non‐terminal event of interest (e.g., disease onset), but not vice versa. Proceeding from the illness‐death model that is described by the transitions between the states “healthy,” “diseased,” and “death,” we used an AFT approach to model the age‐at‐event variables for the different transitions. The resulting trivariate model incorporates interval censoring for the age at disease onset, right censoring for the age at the death as well as left truncation to account for the delayed entry into the study cohort. We consider a Markovian type model, allowing to additionally truncate the age at death following a diagnosis. Finally, it is possible to include various covariates and to account for potential dependencies between the age at the disease onset and age at death through incorporating random effects.

The introduced model was illustrated using data from the PAQUID study, yielding plausible results and confirming similar findings by other existing methods. In addition, by using parametric assumptions for the three age‐at‐event distributions, we could estimate diverse patient profiles and further investigate the expected age at disease onset, age at death, and age at death after diagnosis depending on different patient characteristics. We assessed the performance of our approach by conducting a simulation study and found promising results that demonstrate very good performance in terms of median bias and MSE for the majority of the model estimates and scenarios.

The major advantage of the model is its intuitive and straightforward interpretation based on the survival instead of the hazard function, thus facilitating communication of results. In addition, estimating and comparing patient profiles can provide more insights into how different risk factors may influence the age at disease onset and the age at death. Furthermore, it is possible to transform the estimated model parameters, allowing the results to be reported as hazard ratios. It is still recommended that acceleration factors be used; however, the option of reporting them on the hazard scale is also provided, should this be preferred for the practical implementation of the model.

Of course, there are some limitations to our approach. First, due to the complexity of the likelihood function that incorporates two types of censoring and left truncation, numerical problems might occur. If such a problem arises, we recommend using the simplified version of the model applied in the simulation study, instead of the more complex model assuming three different variances. As the conducted simulations demonstrated, this version of the model performs similarly to the complex model.

Second, the model is sensitive to the number of events observed for the single transitions. The conducted simulation study indicates that in particular transitions with fewer events, some estimated parameters demonstrate slightly higher MSE. Therefore, we emphasize that an application of the model to severely censored data sets might lead to estimates with larger standard errors.

Third, our model is based on the premise that each transition follows a Weibull distribution. Despite the flexibility of the Weibull distribution, this assumption could be overly restrictive and may not adequately represent all data. Hence, incorporating other parametric assumptions should be considered for future research as it has been demonstrated that the Gompertz distribution might be a better choice for modeling mortalities [28].

Finally, the inclusion of random effects with a complex covariance structure might cause problems in the likelihood maximization in single cases. In particular, the simulation study has demonstrated that the random effects estimation and the respective standard errors may be challenging. Even though the regression parameters of the model with the complex covariance structure are estimated more accurately than those of the simplified version, it is not satisfactory that the random effects variance and covariance parameters cannot be estimated properly. This could be subject to future work on extending the model by using multiple copula functions for flexible modeling of the joint distributions and related dependencies.

Furthermore, for the practical application of the model and easier implementation, we highly recommend fitting three univariate models and using their estimates as initial values for the final model. This way, numerical problems may be easily avoided. In addition, the choice of initial values for the random effects variances and covariances could be essential. We suggest using smaller initial values closer to 0 to possibly avoid numerical problems. Lastly, we recommend always fitting both models discussed in the paper and comparing their performance via model selection criteria, as also done in the real application section. However, despite the mentioned limitations, we provide a new method that can model survival data with complex censoring patterns and dependence structures that often occur in cohort studies. The model's application can be extended to other chronic diseases, contributing to a more precise estimation of their true underlying distributions. Incorporating diverse risk factors and comparing mortalities for diagnosed and disease‐free individuals should provide a more profound understanding of a disease's impact on life expectancy.

## Author Contributions

**Antoniya Dineva:** methodology, formal analysis, software, writing – original draft, writing – review and editing. **Oliver Kuss:** writing – review and editing. **Annika Hoyer:** conceptualization, methodology, writing – review and editing, supervision.

## Funding

The authors have nothing to report.

## Conflicts of Interest

The authors declare no conflicts of interest.

## Supporting information


**Data S1:** sim70405‐sup‐0001‐Supinfo.zip.

## Data Availability

The data that support the findings of this study are openly available in Zenodo at https://zenodo.org/records/17652004.
