# Supplementary material for: A New Parametric Accelerated Failure Time Model for Semi‐Competing Risks Data
Source: Stat Med. 2026 Apr 6;45(8-9):e70405. doi: 10.1002/sim.70405 (PMC13051532; doi:10.1002/sim.70405)
Supplement: Supplementary file 1 — Data S1: sim70405‐sup‐0001‐Supinfo.zip. [file SIM-45-0-s001.zip › sim70405-sup-0001-Supinfo/'Supplementary material.pdf]

APPENDIX

A SUPPLEMENTARY TABLES

A.1 Supplementary information on the simulation study.

Table A1 Final sample size (N), mean number of non-terminal events (n_i) and mean number of terminal events (n_{m_0}, n_{m_1}) in the respective transition over 1000 iterations and for each simulation scenario.

	L (N=2352)			M (N=1176)			S (N=490)		
**Setting**	$\mathbf{n}_i$	$\mathbf{n}_{m_0}$	$\mathbf{n}_{m_1}$	$\mathbf{n}_i$	$\mathbf{n}_{m_0}$	$\mathbf{n}_{m_1}$	$\mathbf{n}_i$	$\mathbf{n}_{m_0}$	$\mathbf{n}_{m_1}$
E65	659	1054	610	330	527	306	138	219	127
E80	659	1053	337	329	527	168	137	219	70
L65	817	1435	811	410	716	407	170	299	169
L80	819	1432	696	410	716	349	170	299	145

Table A2 Mean censoring rates in the incidence transition (c_i) and the two mortality transitions (c_{m_0}, c_{m_1}) over 1000 iterations and for each simulation scenario.

	L			M			S		
**Setting**	$\mathbf{n}_i$	$\mathbf{n}_{m_0}$	$\mathbf{n}_{m_1}$	$\mathbf{n}_i$	$\mathbf{n}_{m_0}$	$\mathbf{n}_{m_1}$	$\mathbf{n}_i$	$\mathbf{n}_{m_0}$	$\mathbf{n}_{m_1}$
E65	72%	55%	74%	72%	55%	74%	71%	55%	74%
E80	72%	55%	86%	72%	55%	86%	71%	55%	86%
L65	65%	39%	65%	65%	39%	65%	65%	39%	65%
L80	65%	39%	70%	65%	39%	70%	65%	39%	70%

A.2 Supplementary tables of model application on simulation study data.

Table A3 True parameter values for data simulation.

Parameter	Setting	True value
s_i^2	all	0.008237
$s_{m_0}^2$	all	0.000714
$s_{m_1}^2$	all	0.00063
c_{i,m_1}	all	0.000115
κ_i	all	8.9461
κ_{m_0}	all	10.3388
κ_{m_1}	all	9.2104
$\beta_{0,i}$	all	4.582482
β_{0,m_0}	all	4.534187
β_{0,m_1}	E80 and L80	4.509869
β_{0,m_1}	E65 and L65	4.30223
$\beta_{1,i}$	all	0.02616
β_{1,m_0}	all	−0.04306
β_{1,m_1}	all	−0.0743
E_i (Female)	all	92.54702
E_{m_0} (Female)	all	88.74005
E_{m_1} (Female)	E65 and L65	70.01974
E_{m_1} (Female)	E80 and L80	86.18
E_i (Male)	all	95.00
E_{m_0} (Male)	all	85.00
E_{m_1} (Male)	E65 and L65	65.00
E_{m_1} (Male)	E80 and L80	80.00
Med_i (Female)	all	93.83270
Med_{m_0} (Female)	all	89.90352
Med_{m_1} (Female)	E65 and L65	70.98273
Med_{m_1} (Female)	E80 and L80	87.36337
Med_i (Male)	all	96.31975
Med_{m_0} (Male)	all	86.11444
Med_{m_1} (Male)	E65 and L65	65.89395
Med_{m_1} (Male)	E80 and L80	81.10025

Table A4 Number of converged runs per 1000 iterations for each simulation setting and model.

Setting	Converged runs model HomVar	Converged runs model HetVar
E65 L	1000	1000
E65 M	1000	1000
E65 S	1000	1000
E80 L	1000	1000
E80 M	1000	1000
E80 S	1000	1000
L65 L	1000	1000
L65 M	1000	1000
L65 S	1000	1000
L80 L	1000	1000
L80 M	1000	1000
L80 S	1000	1000

Table A5 Model HomVar. Median bias and mean squared error (MSE) over 1000 iterations for each simulations scenario: E65, E80, L65, L80, and each initial sample size n=2400, n=1200, and n= 500.

		**n=2400**		**n=1200**		**n=500**	
Setting	Parameter	Median bias	MSE	Median bias	MSE	Median bias	MSE
E65	b_{0,m_1}	0.01	0.00	0.01	0.00	0.02	0.00
E65	b_{0,m_0}	0.00	0.00	0.00	0.00	0.00	0.00
E65	$b_{0,i}$	0.01	0.00	0.00	0.00	0.01	0.00
E65	b_{1,m_1}	-0.01	0.00	-0.01	0.00	-0.01	0.00
E65	b_{1,m_0}	0.00	0.00	0.00	0.00	0.00	0.00
E65	$b_{1,i}$	0.00	0.00	0.00	0.00	0.00	0.00
E65	κ_{m_1}	0.95	1.38	0.93	1.78	0.94	3.13
E65	κ_{m_0}	0.25	0.24	0.23	0.43	0.20	1.00
E65	κ_i	1.09	1.44	1.11	1.71	1.06	2.33
E80	b_{0,m_1}	0.00	0.00	0.00	0.00	0.00	0.00
E80	b_{0,m_0}	0.00	0.00	0.00	0.00	0.00	0.00
E80	$b_{0,i}$	0.01	0.00	0.01	0.00	0.01	0.00
E80	b_{1,m_1}	0.00	0.00	0.00	0.00	0.00	0.00
E80	b_{1,m_0}	0.00	0.00	0.00	0.00	0.00	0.00
E80	$b_{1,i}$	0.00	0.00	0.00	0.00	0.00	0.00
E80	κ_{m_1}	0.14	1.28	0.07	2.67	-0.04	7.60
E80	κ_{m_0}	0.32	0.29	0.30	0.49	0.23	1.22
E80	κ_i	0.92	1.09	0.88	1.28	0.80	1.93
L65	b_{0,m_1}	0.03	0.00	0.03	0.00	0.03	0.00
L65	b_{0,m_0}	0.00	0.00	0.00	0.00	0.00	0.00
L65	$b_{0,i}$	0.00	0.00	0.00	0.00	0.00	0.00
L65	b_{1,m_1}	-0.01	0.00	-0.01	0.00	-0.01	0.00
L65	b_{1,m_0}	0.00	0.00	0.00	0.00	0.00	0.00
L65	$b_{1,i}$	0.00	0.00	0.00	0.00	0.00	0.00
L65	κ_{m_1}	1.42	2.21	1.36	2.38	1.35	3.00
L65	κ_{m_0}	0.35	0.24	0.32	0.33	0.31	0.68
L65	κ_i	1.33	1.89	1.32	2.05	1.25	2.35
L80	b_{0,m_0}	-0.01	0.00	-0.01	0.00	0.00	0.00
L80	$b_{0,i}$	0.00	0.00	0.00	0.00	0.00	0.00
L80	b_{1,m_1}	0.00	0.00	0.00	0.00	0.00	0.00
L80	b_{1,m_0}	0.00	0.00	0.00	0.00	0.00	0.00
L80	$b_{1,i}$	0.00	0.00	0.00	0.00	0.00	0.00
L80	κ_{m_1}	0.03	0.54	-0.05	1.33	-0.19	3.75
L80	κ_{m_0}	0.46	0.33	0.42	0.47	0.30	0.88
L80	κ_i	1.16	1.50	1.13	1.59	1.09	2.04

Table A6 Model HomVar. Median bias and mean squared error (MSE) for the respective mean and median age at the event over 1000 iterations for each simulations scenario: E65, E80, L65, L80, and each initial sample size n=2400, n=1200, and n= 500.

		**n=2400**		**n=1200**		**n=500**	
Setting	Parameter	Median bias	MSE	Median bias	MSE	Median bias	MSE
E65	E_{m_1} (Female)	1.34	5.00	1.30	7.86	1.43	17.60
E65	E_{m_1} (Male)	0.88	4.73	0.85	8.47	0.93	20.14
E65	E_{m_0} (Female	-0.09	0.30	-0.07	0.59	-0.11	1.40
E65	E_{m_0} (Male)	-0.02	0.29	-0.01	0.59	0.00	1.43
E65	E_i(Female)	1.05	1.77	1.02	2.42	1.04	4.44
E65	E_i(Male)	1.10	2.68	1.11	4.45	1.05	9.29
E65	Med_{m_1} (Female)	1.33	5.00	1.29	7.89	1.41	17.80
E65	Med_{m_1} (Male)	0.87	4.75	0.83	8.54	0.91	20.43
E65	Med_{m_0} (Female)	-0.10	0.31	-0.08	0.61	-0.12	1.46
E65	Med_{m_0} (Male)	-0.04	0.29	-0.02	0.60	0.00	1.45
E65	Med_i(Female)	1.03	1.75	0.99	2.42	1.04	4.52
E65	Med_i(Male)	1.07	2.68	1.08	4.51	1.02	9.53
E80	E_{m_1} (Female)	0.11	1.84	-0.07	3.86	-0.21	10.73
E80	E_{m_1} (Male)	0.11	3.29	0.06	7.04	-0.09	19.40
E80	E_{m_0} (Female	-0.22	0.36	-0.21	0.63	-0.22	1.46
E80	E_{m_0} (Male)	-0.10	0.30	-0.09	0.59	-0.10	1.44
E80	E_i(Female)	1.11	1.84	1.09	2.44	1.21	4.48
E80	E_i(Male)	1.21	2.88	1.22	4.23	1.27	8.75
E80	Med_{m_1} (Female)	0.11	1.81	-0.04	3.81	-0.18	10.76
E80	Med_{m_1} (Male)	0.11	3.25	0.07	6.99	-0.08	19.54
E80	Med_{m_0} (Female)	-0.24	0.38	-0.23	0.66	-0.23	1.52
E80	Med_{m_0} (Male)	-0.12	0.31	-0.11	0.61	-0.11	1.47
E80	Med_i(Female)	1.08	1.82	1.08	2.45	1.20	4.59
E80	Med_i(Male)	1.20	2.89	1.22	4.31	1.26	9.01
L65	E_{m_1} (Female)	2.52	8.83	2.39	10.95	2.33	18.16
L65	E_{m_1} (Male)	1.88	6.14	1.69	8.82	1.73	17.72
L65	E_{m_0} (Female	-0.13	0.23	-0.16	0.46	-0.09	1.02
L65	E_{m_0} (Male)	-0.08	0.25	-0.05	0.49	-0.08	1.24
L65	E_i(Female)	0.75	1.09	0.82	1.73	0.84	3.25
L65	E_i(Male)	0.85	2.02	0.89	3.34	0.82	6.99
L65	Med_{m_1} (Female)	2.52	8.85	2.38	11.01	2.33	18.39
L65	Med_{m_1} (Male)	1.87	6.15	1.68	8.89	1.72	17.99
L65	Med_{m_0} (Female)	-0.15	0.24	-0.18	0.47	-0.09	1.04
L65	Med_{m_0} (Male)	-0.10	0.25	-0.06	0.50	-0.09	1.25
L65	Med_i(Female)	0.72	1.05	0.79	1.70	0.80	3.28
L65	Med_i(Male)	0.81	2.00	0.85	3.36	0.80	7.15
L80	E_{m_1} (Female)	0.02	1.41	-0.07	3.14	-0.27	7.20
L80	E_{m_1} (Male)	-0.02	2.00	-0.19	4.59	-0.32	10.45
L80	E_{m_0} (Female	-0.30	0.30	-0.31	0.53	-0.24	1.14
L80	E_{m_0} (Male)	-0.16	0.29	-0.17	0.53	-0.14	1.20
L80	E_i(Female)	0.75	1.08	0.79	1.66	0.84	3.08
L80	E_i(Male)	0.91	2.01	0.97	3.24	0.92	6.79
L80	Med_{m_1} (Female)	0.01	1.38	-0.07	3.07	-0.28	7.04
L80	Med_{m_1} (Male)	-0.02	1.98	-0.18	4.55	-0.31	10.33
L80	Med_{m_0} (Female)	-0.33	0.32	-0.34	0.55	-0.25	1.18
L80	Med_{m_0} (Male)	-0.18	0.30	-0.19	0.55	-0.16	1.21
L80	Med_i(Female)	0.72	1.05	0.76	1.65	0.81	3.12
L80	Med_i(Male)	0.88	2.00	0.94	3.27	0.89	6.96

Table A7 Model HetVar. Median bias and mean squared error (MSE) over 1000 iterations for each simulations scenario: E65, E80, L65, L80, and each initial sample size n=2400, n=1200, and n=500.

		**n=2400**		**n=1200**		**n=500**	
Setting	Parameter	Median bias	MSE	Median bias	MSE	Median Bias	MSE
E65	b_{0,m_1}	0.01	0.00	0.01	0.00	0.02	0.00
E65	b_{0,m_0}	0.00	0.00	0.00	0.00	0.00	0.00
E65	$b_{0,i}$	0.01	0.00	0.00	0.00	0.01	0.00
E65	b_{1,m_1}	-0.01	0.00	-0.01	0.00	-0.01	0.00
E65	b_{1,m_0}	0.00	0.00	0.00	0.00	0.00	0.00
E65	$b_{1,i}$	0.00	0.00	0.00	0.00	0.00	0.00
E65	κ_{m_1}	0.95	1.36	0.94	1.74	0.94	3.02
E65	κ_{m_0}	0.13	0.17	0.12	0.32	0.09	0.78
E65	κ_i	1.09	1.42	1.11	1.68	1.06	2.20
E65	s_i^2	0.01	-	0.01	-	0.01	-
E65	$s_{m_0}^2$	0.00	-	0.00	-	0.00	-
E65	$s_{m_1}^2$	0.00	-	0.00	-	0.00	-
E65	c_{i,m_1}	-4.00	-	-4.00	-	-4.00	-
E65	c_{m_0,m_1}	0.00	-	0.00	-	0.00	-
E80	b_{0,m_1}	0.00	0.00	0.00	0.00	0.00	0.00
E80	b_{0,m_0}	0.00	0.00	0.00	0.00	0.00	0.00
E80	$b_{0,i}$	0.01	0.00	0.01	0.00	0.01	0.00
E80	b_{1,m_1}	0.00	0.00	0.00	0.00	0.00	0.00
E80	b_{1,m_0}	0.00	0.00	0.00	0.00	0.00	0.00
E80	$b_{1,i}$	0.00	0.00	0.00	0.00	0.00	0.00
E80	κ_{m_1}	0.14	1.20	0.07	2.44	0.06	6.00
E80	κ_{m_0}	0.16	0.20	0.15	0.36	0.11	0.80
E80	κ_i	0.92	1.07	0.89	1.25	0.83	1.88
E80	s_i^2	0.01	-	0.01	-	0.01	-
E80	$s_{m_0}^2$	0.00	-	0.00	-	0.00	-
E80	$s_{m_1}^2$	0.00	-	0.00	-	0.00	-
E80	c_{i,m_1}	-4.00	-	-4.00	-	-4.00	-
E80	c_{m_0,m_1}	0.00	-	0.00	-	0.00	-
L65	b_{0,m_1}	0.03	0.00	0.03	0.00	0.03	0.00
L65	b_{0,m_0}	0.00	0.00	0.00	0.00	0.00	0.00
L65	$b_{0,i}$	0.00	0.00	0.00	0.00	0.00	0.00
L65	b_{1,m_1}	-0.01	0.00	-0.01	0.00	-0.01	0.00
L65	b_{1,m_0}	0.00	0.00	0.00	0.00	0.00	0.00
L65	$b_{1,i}$	0.00	0.00	0.00	0.00	0.00	0.00
L65	κ_{m_1}	1.42	2.19	1.37	2.35	1.36	2.97
L65	κ_{m_0}	0.22	0.15	0.19	0.23	0.19	0.49
L65	κ_i	1.33	1.88	1.32	2.03	1.26	2.29
L65	s_i^2	0.01	-	0.01	-	0.01	-
L65	$s_{m_0}^2$	0.00	-	0.00	-	0.00	-
L65	$s_{m_1}^2$	0.00	-	0.00	-	0.00	-
L65	c_{i,m_1}	-4.00	-	-4.00	-	-4.00	-
L65	c_{m_0,m_1}	0.00	-	0.00	-	0.00	-
L80	b_{0,m_1}	0.00	0.00	0.00	0.00	0.00	0.00
L80	b_{0,m_0}	0.00	0.00	0.00	0.00	0.00	0.00
L80	$b_{0,i}$	0.00	0.00	0.00	0.00	0.00	0.00
L80	b_{1,m_1}	0.00	0.00	0.00	0.00	0.00	0.00
L80	b_{1,m_0}	0.00	0.00	0.00	0.00	0.00	0.00
L80	$b_{1,i}$	0.00	0.00	0.00	0.00	0.00	0.00
L80	κ_{m_1}	0.08	0.43	0.06	0.91	0.03	2.21
L80	κ_{m_0}	0.28	0.18	0.28	0.28	0.22	0.49
L80	κ_i	1.18	1.54	1.15	1.64	1.19	2.07
L80	s_i^2	0.01	-	0.01	-	0.01	-
L80	$s_{m_0}^2$	0.00	-	0.00	-	0.00	-
L80	$s_{m_1}^2$	0.00	-	0.00	-	0.00	-
L80	c_{i,m_1}	-4.00	-	-4.00	-	-4.00	-
L80	c_{m_0,m_1}	0.00	-	0.00	-	0.00	-

Table A8 Model HetVar. Median bias and mean squared error (MSE) for the mean and median age at the event over 1000 iterations for each simulations scenario: E65, E80, L65, L80, and each initial sample size n=2400, n=1200, and n=500.

		n=2400		n=1200		n=500	
Setting	Parameter	Median bias	MSE	Median bias	MSE	Median Bias	MSE
E65	E_{m_1}(Female)	1.34	4.86	1.30	7.60	1.43	16.90
E65	E_{m_1}(Male)	0.88	4.54	0.85	8.11	0.96	19.16
E65	E_{m_0}(Female	0.18	0.31	0.21	0.59	0.16	1.34
E65	E_{m_0}(Male)	0.13	0.29	0.15	0.58	0.16	1.40
E65	E_i(Female)	1.05	1.76	1.02	2.39	1.04	4.35
E65	E_i(Male)	1.10	2.65	1.11	4.37	1.05	9.08
E65	Med_{m_1}(Female)	1.33	4.86	1.29	7.63	1.41	17.10
E65	Med_{m_1}(Male)	0.87	4.55	0.84	8.18	0.94	19.43
E65	Med_{m_0}(Female)	0.18	0.32	0.20	0.61	0.15	1.38
E65	Med_{m_0}(Male)	0.12	0.29	0.15	0.59	0.17	1.42
E65	Med_i(Female)	1.03	1.74	0.99	2.39	1.03	4.43
E65	Med_i(Male)	1.07	2.65	1.08	4.43	1.02	9.31
E80	E_{m_1}(Female)	0.12	1.66	-0.05	3.39	-0.02	9.11
E80	E_{m_1}(Male)	0.12	2.97	0.07	6.29	0.07	16.86
E80	E_{m_0}(Female	0.11	0.30	0.11	0.55	0.12	1.35
E80	E_{m_0}(Male)	0.11	0.28	0.11	0.56	0.10	1.37
E80	E_i(Female)	1.11	1.82	1.07	2.39	1.18	4.36
E80	E_i(Male)	1.21	2.83	1.22	4.13	1.17	8.43
E80	Med_{m_1}(Female)	0.12	1.63	-0.04	3.34	0.00	9.18
E80	Med_{m_1}(Male)	0.11	2.94	0.09	6.25	0.07	17.03
E80	Med_{m_0}(Female)	0.10	0.31	0.11	0.57	0.11	1.40
E80	Med_{m_0}(Male)	0.09	0.29	0.10	0.57	0.09	1.39
E80	Med_i(Female)	1.08	1.80	1.07	2.40	1.18	4.46
E80	Med_i(Male)	1.19	2.84	1.21	4.20	1.16	8.67
L65	E_{m_1}(Female)	2.52	8.60	2.41	10.64	2.37	17.75
L65	E_{m_1}(Male)	1.88	5.84	1.70	8.41	1.75	17.00
L65	E_{m_0}(Female	0.15	0.23	0.14	0.43	0.20	1.00
L65	E_{m_0}(Male)	0.08	0.24	0.11	0.48	0.08	1.18
L65	E_i(Female)	0.75	1.08	0.82	1.69	0.81	3.20
L65	E_i(Male)	0.85	1.99	0.88	3.25	0.80	6.84
L65	Med_{m_1}(Female)	2.52	8.61	2.40	10.70	2.36	17.97
L65	Med_{m_1}(Male)	1.87	5.84	1.68	8.47	1.73	17.26
L65	Med_{m_0}(Female)	0.15	0.23	0.12	0.44	0.21	1.01
L65	Med_{m_0}(Male)	0.07	0.24	0.10	0.48	0.07	1.19
L65	Med_i(Female)	0.72	1.04	0.78	1.67	0.78	3.23
L65	Med_i(Male)	0.81	1.97	0.84	3.27	0.76	7.00
L80	E_{m_1}(Female)	0.09	1.18	0.12	2.58	0.04	6.12
L80	E_{m_1}(Male)	0.05	1.71	0.02	3.92	0.04	9.10
L80	E_{m_0}(Female	0.07	0.21	0.07	0.41	0.09	1.00
L80	E_{m_0}(Male)	0.04	0.25	0.02	0.48	0.03	1.11
L80	E_i(Female)	0.72	1.03	0.76	1.55	0.77	2.89
L80	E_i(Male)	0.87	1.89	0.89	3.00	0.81	6.41
L80	Med_{m_1}(Female)	0.09	1.16	0.12	2.55	0.04	6.05
L80	Med_{m_1}(Male)	0.05	1.70	0.02	3.90	0.05	9.08
L80	Med_{m_0}(Female)	0.05	0.21	0.05	0.42	0.08	1.02
L80	Med_{m_0}(Male)	0.02	0.25	0.02	0.48	0.02	1.12
L80	Med_i(Female)	0.69	1.00	0.72	1.53	0.73	2.92
L80	Med_i(Male)	0.84	1.88	0.86	3.03	0.78	6.56

Table A9 Model HomVar. Coverage (%) over 1000 iterations for each simulations scenario: E65,E80, L65, L80 and each initial sample size n=2400, n=1200 and n= 500.

		**n=2400**	**n=1200**	**n=500**
Setting	Parameter	Coverage	Coverage	Coverage
E65	b_{0,m_1}	90.5	96.7	98.1
E65	b_{0,m_0}	94.1	94.4	95.6
E65	$b_{0,i}$	86.5	91.0	93.0
E65	b_{1,m_1}	94.3	96.5	97.0
E65	b_{1,m_0}	95.3	94.4	93.9
E65	$b_{1,i}$	95.0	94.1	94.8
E65	κ_{m_1}	53.7	75.1	89.1
E65	κ_{m_0}	87.6	92.7	92.6
E65	κ_i	14.0	40.5	73.7
E80	b_{0,m_1}	94.4	97.1	96.4
E80	b_{0,m_0}	87.0	93.8	95.7
E80	$b_{0,i}$	83.5	87.2	90.6
E80	b_{1,m_1}	95.9	96.1	96.9
E80	b_{1,m_0}	94.0	94.5	95.2
E80	$b_{1,i}$	94.9	96.7	95.6
E80	κ_{m_1}	95.3	95.4	95.1
E80	κ_{m_0}	82.1	88.8	93.1
E80	κ_i	28.1	60.2	81.8
L65	b_{0,m_1}	49.6	80.4	96.4
L65	b_{0,m_0}	86.8	90.2	94.4
L65	$b_{0,i}$	94.5	95.3	93.5
L65	b_{1,m_1}	90.8	96.2	98.1
L65	b_{1,m_0}	93.8	94.8	93.8
L65	$b_{1,i}$	94.8	93.9	94.1
L65	κ_{m_1}	2.1	22.0	64.4
L65	κ_{m_0}	65.7	83.8	90.5
L65	κ_i	0.8	8.5	49.1
L80	b_{0,m_1}	94.8	93.8	93.6
L80	b_{0,m_0}	71.8	85.8	91.0
L80	$b_{0,i}$	93.5	91.9	94.8
L80	b_{1,m_1}	96.1	95.1	94.5
L80	b_{1,m_0}	94.3	93.9	93.7
L80	$b_{1,i}$	94.2	94.6	96.5
L80	κ_{m_1}	95.7	95.1	95.4
L80	κ_{m_0}	53.3	75.6	88.0
L80	κ_i	3.8	25.0	60.6

Table A10 Model HomVar. Coverage (%) for the mean and median age at the event over 1000 iterations for each simulations scenario: E65,E80, L65, L80 and each initial sample size n=2400, n=1200, and n= 500.

		**n=2400**	**n=1200**	**n=500**
Setting	Parameter	Coverage	Coverage	Coverage
E65	E_{m_1}(Female)	87.5	95.0	97.5
E65	E_{m_1}(Male)	94.1	97.2	97.9
E65	E_{m_0}(Female	94.3	95.0	96.4
E65	E_{m_0}(Male)	95.6	95.3	94.3
E65	E_i(Female)	56.0	75.3	83.5
E65	E_i(Male)	74.7	81.1	87.2
E65	Med_{m_1}(Female)	87.7	95.4	97.7
E65	Med_{m_1}(Male)	94.3	97.3	97.9
E65	Med_{m_0}(Female)	94.3	95.0	96.3
E65	Med_{m_0}(Male)	95.3	95.2	94.3
E65	Med_i(Female)	59.5	76.5	84.5
E65	Med_i(Male)	75.7	82.3	87.7
E80	E_{m_1}(Female)	95.3	96.5	94.0
E80	E_{m_1}(Male)	95.8	96.0	93.8
E80	E_{m_0}(Female	89.9	95.5	95.9
E80	E_{m_0}(Male)	93.6	93.8	94.8
E80	E_i(Female)	51.5	73.1	80.6
E80	E_i(Male)	69.7	81.9	85.3
E80	Med_{m_1}(Female)	95.5	97.0	94.2
E80	Med_{m_1}(Male)	95.8	96.2	93.8
E80	Med_{m_0}(Female)	89.0	95.1	95.8
E80	Med_{m_0}(Male)	93.7	94.2	94.9
E80	Med_i(Female)	54.5	74.3	81.6
E80	Med_i(Male)	70.7	82.5	85.9
L65	E_{m_1}(Female)	36.7	74.3	93.3
L65	E_{m_1}(Male)	75.2	90.7	97.2
L65	E_{m_0}(Female	93.7	92.4	94.8
L65	E_{m_0}(Male)	95.5	95.0	95.3
L65	E_i(Female)	67.3	77.7	86.2
L65	E_i(Male)	77.9	84.6	89.4
L65	Med_{m_1}(Female)	37.8	74.8	93.9
L65	Med_{m_1}(Male)	75.8	91.0	97.5
L65	Med_{m_0}(Female)	92.9	92.6	94.9
L65	Med_{m_0}(Male)	95.4	95.2	95.1
L65	Med_i(Female)	70.9	78.6	86.8
L65	Med_i(Male)	79.2	85.4	89.9
L80	E_{m_1}(Female)	94.7	94.3	93.2
L80	E_{m_1}(Male)	96.2	94.3	93.7
L80	E_{m_0}(Female	86.2	90.6	93.2
L80	E_{m_0}(Male)	92.9	94.2	95.6
L80	E_i(Female)	68.0	79.3	87.5
L80	E_i(Male)	75.7	85.5	89.4
L80	Med_{m_1}(Female)	94.7	94.2	93.2
L80	Med_{m_1}(Male)	96.1	94.3	93.6
L80	Med_{m_0}(Female)	84.8	90.4	93.0
L80	Med_{m_0}(Male)	91.8	93.8	95.3
L80	Med_i(Female)	70.5	80.9	87.9
L80	Med_i(Male)	77.0	86.2	89.7

Table A11 Model HetVar. Coverage (%) over 1000 iterations for each simulations scenario: E65,E80, L65, L80 and each initial sample size n=2400, n=1200 and n= 500.

		n=2400	n=1200	n=500
Setting	Parameter	Coverage	Coverage	Coverage
E65	b_{0,m_1}	88.2	94.8	97.0
E65	b_{0,m_0}	90.8	92.8	94.0
E65	$b_{0,i}$	84.9	90.2	91.3
E65	b_{1,m_1}	90.3	92.7	93.9
E65	b_{1,m_0}	95.0	93.9	93.7
E65	$b_{1,i}$	94.7	93.0	94.4
E65	κ_{m_1}	51.0	71.7	86.9
E65	κ_{m_0}	79.1	81.4	79.0
E65	κ_i	9.6	35.5	63.9
E65	s_i^2	-	-	-
E65	$s_{m_0}^2$	-	-	-
E65	$s_{m_1}^2$	-	-	-
E65	c_{i,m_1}	-	-	-
E65	c_{m_0,m_1}	-	-	-
E80	b_{0,m_1}	89.4	92.0	92.1
E80	b_{0,m_0}	92.6	93.9	93.8
E80	$b_{0,i}$	81.6	86.2	90.1
E80	b_{1,m_1}	90.8	90.6	92.5
E80	b_{1,m_0}	93.6	94.4	94.6
E80	$b_{1,i}$	94.8	95.6	95.4
E80	κ_{m_1}	92.5	92.5	93.2
E80	κ_{m_0}	86.7	87.0	86.2
E80	κ_i	22.9	54.8	76.5
E80	s_i^2	-	-	-
E80	$s_{m_0}^2$	-	-	-
E80	$s_{m_1}^2$	-	-	-
E80	c_{i,m_1}	-	-	-
E80	c_{m_0,m_1}	-	-	-
L65	b_{0,m_1}	38.8	73.5	93.3
L65	b_{0,m_0}	94.4	93.8	94.2
L65	$b_{0,i}$	93.5	94.6	93.1
L65	b_{1,m_1}	80.3	91.3	93.6
L65	b_{1,m_0}	93.6	94.0	93.6
L65	$b_{1,i}$	94.0	93.0	93.7
L65	κ_{m_1}	1.5	16.7	58.4
L65	κ_{m_0}	74.4	84.0	80.5
L65	κ_i	0.3	5.9	39.6
L65	s_i^2	-	-	-
L65	$s_{m_0}^2$	-	-	-
L65	$s_{m_1}^2$	-	-	-
L65	c_{i,m_1}	-	-	-
L65	c_{m_0,m_1}	-	-	-
L80	b_{0,m_1}	90.2	90.7	93.3
L80	b_{0,m_0}	94.5	93.7	94.1
L80	$b_{0,i}$	92.9	91.4	94.4
L80	b_{1,m_1}	88.9	87.6	89.8
L80	b_{1,m_0}	94.3	93.6	92.9
L80	$b_{1,i}$	93.6	93.3	95.9
L80	κ_{m_1}	93.0	92.7	94.6
L80	κ_{m_0}	72.8	78.6	81.8
L80	κ_i	1.3	14.1	43.7
L80	s_i^2	-	-	-
L80	$s_{m_0}^2$	-	-	-
L80	$s_{m_1}^2$	-	-	-
L80	c_{i,m_1}	-	-	-
L80	c_{m_0,m_1}	-	-	-

Table A12 Model HetVar. Coverage (%) for the mean and median age at the event over 1000 iterations for each simulations scenario: E65,E80, L65, L80 and each initial sample size n=2400, n=1200, and n= 500.

		**n=2400**	**n=1200**	**n=500**
Setting	Parameter	Coverage	Coverage	Coverage
E65	E_{m_1}(Female)	84.5	93.0	95.9
E65	E_{m_1}(Male)	92.4	95.9	97.1
E65	E_{m_0}(Female	90.1	92.3	94.7
E65	E_{m_0}(Male)	93.4	94.6	93.1
E65	E_i(Female)	55.2	74.3	82.5
E65	E_i(Male)	73.3	79.9	86.6
E65	Med_{m_1}(Female)	85.2	93.4	96.0
E65	Med_{m_1}(Male)	92.5	96.2	97.1
E65	Med_{m_0}(Female)	90.5	92.6	94.5
E65	Med_{m_0}(Male)	93.4	94.8	93.1
E65	Med_i(Female)	57.8	75.4	83.6
E65	Med_i(Male)	74.5	80.9	87.1
E80	E_{m_1}(Female)	90.8	92.4	90.5
E80	E_{m_1}(Male)	92.2	92.1	93.4
E80	E_{m_0}(Female	92.1	94.0	93.8
E80	E_{m_0}(Male)	94.1	94.1	94.4
E80	E_i(Female)	50.3	70.8	80.6
E80	E_i(Male)	68.5	81.0	85.1
E80	Med_{m_1}(Female)	90.7	92.6	90.3
E80	Med_{m_1}(Male)	92.2	92.0	93.3
E80	Med_{m_0}(Female)	92.2	94.4	93.9
E80	Med_{m_0}(Male)	94.0	94.2	94.6
E80	Med_i(Female)	53.2	72.4	81.3
E80	Med_i(Male)	69.7	81.4	85.9
L65	E_{m_1}(Female)	29.3	67.1	89.7
L65	E_{m_1}(Male)	64.4	85.5	94.0
L65	E_{m_0}(Female	90.4	92.8	93.6
L65	E_{m_0}(Male)	93.0	93.6	93.9
L65	E_i(Female)	66.2	75.7	85.3
L65	E_i(Male)	76.7	83.2	89.0
L65	Med_{m_1}(Female)	29.8	67.9	90.3
L65	Med_{m_1}(Male)	65.2	85.8	94.2
L65	Med_{m_0}(Female)	91.0	93.1	93.8
L65	Med_{m_0}(Male)	93.8	93.6	94.5
L65	Med_i(Female)	69.8	77.4	86.3
L65	Med_i(Male)	77.7	83.6	89.3
L80	E_{m_1}(Female)	90.6	92.0	93.7
L80	E_{m_1}(Male)	92.0	91.1	92.8
L80	E_{m_0}(Female	94.1	94.3	93.1
L80	E_{m_0}(Male)	93.3	93.2	94.8
L80	E_i(Female)	66.5	79.4	88.2
L80	E_i(Male)	74.6	85.0	90.5
L80	Med_{m_1}(Female)	90.8	91.8	93.4
L80	Med_{m_1}(Male)	91.8	90.9	92.9
L80	Med_{m_0}(Female)	94.0	94.1	93.4
L80	Med_{m_0}(Male)	93.4	93.1	94.7
L80	Med_i(Female)	70.0	80.9	88.4
L80	Med_i(Male)	76.4	86.0	90.7
